# Neuroendocrine pathways and breast cancer progression: a pooled analysis of somatic mutations and gene expression from two large breast cancer cohorts

**DOI:** 10.1186/s12885-022-09779-8

**Published:** 2022-06-21

**Authors:** Kejia Hu, Chengshi Wang, Chuanxu Luo, Hong Zheng, Huan Song, Jacob Bergstedt, Katja Fall, Ting Luo, Kamila Czene, Unnur A. Valdimarsdóttir, Fang Fang, Donghao Lu

**Affiliations:** 1 Laboratory of Molecular Diagnosis of Cancer, Clinical Research Center for Breast, West China Hospital, Sichuan University, Chengdu, China; 2 Unit of Integrative Epidemiology, Institute of Environmental Medicine, Karolinska Institutet, Stockholm, Sweden; 3 Sichuan Cancer Hospital & Institute, Sichuan Cancer Center, School of Medicine, University of Electronic Science and Technology of China, Chengdu, China; 4 West China Biomedical Big Data Center, West China Hospital, Sichuan University, Chengdu, China; 5 Center of Public Health Sciences, Faculty of Medicine, University of Iceland, Reykjavík, Iceland; 6 Department of Medical Epidemiology and Biostatistics, Karolinska Institutet, Stockholm, Sweden; 7 Clinical Epidemiology and Biostatistics, School of Medical Sciences, Örebro Universitet, Örebro, Sweden; 8 Department of Epidemiology, Harvard T.H. Chan School of Public Health, Boston, MA USA

**Keywords:** Breast cancer, Somatic mutation, Differential expression, Pathway

## Abstract

**Background:**

Experimental studies indicate that neuroendocrine pathways might play a role in progression of breast cancer. We aim to test the hypothesis that somatic mutations in the genes of neuroendocrine pathways influence breast cancer prognosis, through dysregulated gene expression in tumor tissue.

**Methods:**

We conducted an extreme case–control study including 208 breast cancer patients with poor invasive disease-free survival (iDFS) and 208 patients with favorable iDFS who were individually matched on molecular subtype from the Breast Cancer Cohort at West China Hospital (WCH; *N* = 192) and The Cancer Genome Atlas (TCGA; *N* = 224). Whole exome sequencing and RNA sequencing of tumor and paired normal breast tissues were performed. Adrenergic, glucocorticoid, dopaminergic, serotonergic, and cholinergic pathways were assessed for differences in mutation burden and gene expression in relation to breast cancer iDFS using the logistic regression and global test, respectively.

**Results:**

In the pooled analysis, presence of any somatic mutation (odds ratio = 1.66, 95% CI: 1.07–2.58) of the glucocorticoid pathway was associated with poor iDFS and a two-fold increase of tumor mutation burden was associated with 17% elevated odds (95% CI: 2–35%), after adjustment for cohort membership, age, menopausal status, molecular subtype, and tumor stage. Differential expression of genes in the glucocorticoid pathway in tumor tissue (*P* = 0.028), but not normal tissue (*P* = 0.701), was associated with poor iDFS. Somatic mutation of the adrenergic and cholinergic pathways was significantly associated with iDFS in WCH, but not in TCGA.

**Conclusion:**

Glucocorticoid pathway may play a role in breast cancer prognosis through differential mutations and expression. Further characterization of its functional role may open new avenues for the development of novel therapeutic targets for breast cancer.

**Supplementary Information:**

The online version contains supplementary material available at 10.1186/s12885-022-09779-8.

## Background

Globally, breast cancer is the most common cancer in women [1]. Although the survival rate has improved substantially over the past decades, breast cancer remains the leading cause of cancer death among women [1] and one third of patients with early-stage breast cancer will ultimately develop a metastatic disease [2]. To date, estrogen receptor (ER), progesterone receptor (PR), and human epidermal growth factor receptor 2 (HER2) are the most well-established biomarkers to predict prognosis and guide treatment [3]. However, variable clinical courses are not completely captured by these biomarkers [4], and not all tumors respond to the targeted therapy [5]. It is therefore important to advance our understanding of the complex biological mechanisms underlying breast cancer progression and to identify new biomarkers.

Emerging evidence suggests that neuroendocrine pathways play a salient role in the development and progression of many cancers, including breast cancer [6–13]. For instance, animal models showed that β-adrenergic pathway activation facilitated breast cancer metastasis [7]. Glucocorticoid pathway has also been suggested to be involved in multiple processes of breast cancer metastasis, including cell adhesion, chemoresistance, evasion of apoptosis, and angiogenesis [8–11]. Furthermore, the blockade of cholinergic receptors reduces the proliferation of breast cancer cells [12, 14]. On the other hand, dopamine inhibits tumor angiogenesis [15] and bone metastasis in breast cancer [16], while serotonin stimulates breast tumor proliferation and apoptosis evasion [17, 18].

Data from human studies are however relatively scarce. Cholinergic pathway signaling was shown to be associated with breast cancer recurrence among ER-negative patients [19]. A high expression level of glucocorticoid receptor and its activation-associated genes correlated with shorter relapse-free survival [9, 10]. We found in our previous studies that molecular signatures of adrenergic, glucocorticoid, and serotoninergic pathways were associated with lethal outcome in patients with prostate cancer [20, 21]. As germline variants of neuroendocrine pathways appeared to only explain a small proportion of the dysregulated signaling [20], somatic mutations in neuroendocrine pathways may drive the differential signaling leading to putative biological effects in cancer progression. We, therefore, undertook an integrative molecular approach by pooling data from two large breast cancer cohorts and tested the hypothesis that somatic mutations in the genes of neuroendocrine pathways influence breast cancer prognosis, through dysregulated gene expression in tumor tissue.

## Methods

### Study populations

The Breast Cancer Cohort at the West China Hospital, Sichuan University, China (the WCH cohort) is a prospective cohort of breast cancer patients diagnosed in the WCH from 2008 onward[22]. As of April 15^th^, 2018, 7,784 women who were diagnosed with non-metastatic invasive disease were included and prospectively followed for clinical outcomes. Complete information on demographic factors, tumor characteristics, and treatment were collected directly from an interview at baseline and the medical records in WCH. The median follow-up was 4.6 years by April 2018 (Q1 = 2.7, Q3 = 6.9 years). Fresh frozen tumor and/or normal breast tissues were collected at primary surgery whereas blood samples were donated at the time of diagnosis. Patients who have donated both tumor and germline tissues (normal breast tissue or blood) were eligible for the present study (*N* = 1,462).

The Cancer Genome Atlas (TCGA) [23] is an international project that molecularly characterized a large sample of multiple cancers, including breast cancer. Clinical and molecular data have been made publicly available at GDC Data Portal (https://portal.gdc.cancer.gov/projects/TCGA-BLCA). Women who were diagnosed as non-metastatic invasive breast adenocarcinoma and had fresh-frozen samples of primary tumor collected at surgical resection prior to other treatment (chemotherapy or radiotherapy) were enrolled during 2009–2015 (*N* = 1,063) and followed since diagnosis. The median follow-up was 2.3 years by 2015 (Q1 = 1.2, Q3 = 4.6 years) [24]. Similarly, patients with available data on somatic mutations were eligible for the study (*N* = 953).

### Matched extreme case–control design

To maximize the statistical power and optimize the cost-effectiveness, we employed a matched extreme case–control design which has been successfully implemented to study prognostic biomarkers of cancers by comparing patients with the worst prognosis to those with the most favorable survival [25]. In the present study, patients with any invasive disease-free survival (iDFS) endpoints during the first five years after cancer diagnosis were identified as cases (i.e., with poor prognosis), whereas patients who survived at least five years after diagnosis and had no iDFS endpoints through the last follow-up were considered as controls (i.e., with favorable prognosis). Any local or regional recurrence, distant metastasis, new primary tumors from any sites, cancer-specific death, and death from other causes were defined as iDFS endpoints [26]. One control per case was randomly selected and individually matched to the case on molecular subtype classified according to St Gallen Consensus 2013 [27] (see details in the Supplementary Methods). To avoid introducing potential selection bias, age at diagnosis was not restricted. Finally, we identified 96 cases and 96 controls from the WCH cohort, and 112 cases and 112 controls from TCGA. Among them, 208 cases and 208 controls with somatic mutation data; of which, 202 cases and 205 controls were sequenced for tumor RNA, and 142 normal breast tissues were also sequenced for RNA in the WCH cohort.

### Neuroendocrine pathway selection and construction

As previously described [20, 21], we focused on genes in five neuroendocrine pathways with a suggested link to psychological distress. We identified the genes in each pathway from the Kyoto Encyclopedia of Genes and Genomes (KEGG) and based on our previous studies [20, 21]. In total, 544 unique genes were identified, including 236 genes for the adrenergic pathway, 153 for the glucocorticoid pathway, 138 for the dopaminergic pathway, 125 for the serotonergic pathway, and 235 for the cholinergic pathway. The full list and information of each pathway are presented in Table S1.

### Whole exome sequencing and RNA-sequencing

In the WCH cohort, DNA was extracted from the tumor tissue and germline-derived samples. Whole Exome Sequencing (WES) was performed on Illumina Novaseq S6000 platform. After quality control, reads were mapped to the reference genome (UCSC hg38) using Burrows-Wheeler Aligner (BWA) software [28]. In TCGA cohort, WES was performed for tumor tissue on Illumina Hi-Seq 2000 platform. Somatic mutation data were downloaded from GDC portal.

In the WCH cohort, RNA sequencing for frozen tumor and normal breast tissue was performed on the Illumina Novaseq S6000 platform. After quality control, reads were mapped to reference genome using Hisat2 v2.0.5 [29]. In TCGA, Raw read counts produced by HT-Seq of tumor RNA were downloaded from the GDC portal. A detailed description of the sample preparation and bioinformatic pipeline for WES and RNA-seq was available in Supplementary methods.

### Statistical analysis

#### Somatic mutations

Tumor mutation burden (TMB) has been established as an important biomarker for cancer survival [30], including breast cancer [31, 32]. Therefore, we calculated somatic TMB for each of the neuroendocrine pathways, defined as the number of mutations in candidate genes per 1 million base pairs of the coding sequence in these genes. TMB was analyzed as a binary variable (any vs. no mutation) and a continuous variable after log2 transformation. Odds ratios (ORs) were estimated using multivariable logistic regression models adjusting for cohort membership, age at diagnosis, menopausal status at diagnosis, molecular subtype, and cancer stage. In addition, we employed a basic model with adjustment for only cohort membership and molecular subtype if one considers that cancer stage is an outline of tumor genome (i.e., mediator) and therefore not necessarily to be adjusted for; and based on the main model, we fitted an advanced model with further adjustment for cancer treatment, which was largely planned according to molecular subtype and tumor characteristics. Estimates from both cohorts were formally compared for heterogeneity using Cochrane’s Q test.

#### Gene expression

We used TMM normalization [33] to normalize the library size of each sample in both cohorts. Counts per million reads (CPM) in a log2 scale were calculated. We adjusted for batch effect using Combat function in sva package [34] when pooling gene expression data from two cohorts. We used the global test [35] to compare the gene expression between cases and controls at the pathway level. To further shed light on the direction of the association, we analyzed associations between TMB of glucocorticoid pathway and individual gene expression using linear regression; and associations between individual gene expression and iDFS endpoints using logistic regression. We also performed a mediation analysis for the top four significant genes, to estimate in what proportion these individual gene expressions mediated the association between TMB of glucocorticoid pathway and iDFS endpoints. Cohort membership, age at diagnosis, menopausal status at diagnosis, molecular subtype, and cancer stage were adjusted for in these analyses.

To assess the role of ER status on these results, we stratified the analyses for somatic mutations and gene expression by ER status. To test the robustness of these results, we performed additional analyses by excluding the matching pairs of cases with non-breast-cancer death or patients who underwent neoadjuvant chemotherapy, and by removing the overlapping genes across the five pathways.

The statistical analyses were performed in R (version 4.1.2). We used a two-sided *P* < 0.05 to indicate statistical significance.

## Results

Compared with the controls (patients with favorable prognosis), the cases (patients with poor prognosis) were older, more likely to be postmenopausal, and had a more advanced stage at diagnosis (Table 1). The ER, PR, HER2 status and molecular subtypes were largely similar between cases and controls, although the patients of unclassified subtype and unknown HER2 status were fewer in controls due to the matching method. In line with the full cohort, all WCH participants were Asian, mostly Han Chinese (*N* = 189; 98%); while in TCGA, the majority were white (*N* = 170; 76%), followed by black or African American (*N* = 35; 16%). Treatment was similar between cases and controls in the WCH sample. In the TCGA sample, fewer cases than controls received chemotherapy, radiotherapy, or hormonal therapy. These patterns were similar between the WCH cohort and TCGA, except for more similar age distribution between cases and controls and more cases with radiotherapy in the WCH cohort (Table S2). Cases/controls nested from two cohorts were comparable in the distribution of cancer stage, although patients in the WCH cohort were younger, had a higher proportion of Luminal B subtype, and had more mastectomy and chemotherapy, which also represented the discrepancies between the whole WCH and TCGA cohorts (Table S2).

**Table 1:** Clinical characteristics of breast cancer patients with poor (cases) and favorable (controls) invasive disease-free survival

	Cases	Controls	*P* value
Number of patients	208	208	
Age at diagnosis			< 0.01^a^
Mean (SD)	56 (15)	52 (11)	
Range	23—90	27—83	
	N (%)	N (%)	
Age at diagnosis			< 0.01^b^
23–39	28 (13.5%)	25 (12.0%)	
40–49	50 (24.0%)	77 (37.0%)	
50–59	51 (24.5%)	57 (27.4%)	
60–69	38 (18.3%)	31 (14.9%)	
70–90	41 (19.7%)	18 (8.7%)	
Menopausal status at diagnosis			< 0.01^b^
Premenopausal	69 (33.2)	101 (48.6)	
Postmenopausal	121 (58.2)	98 (47.1)	
Unknown	18 (8.7)	9 (4.3)	
Stage			< 0.01^b^
Stage I	19 (9.1)	45 (21.6)	
Stage II	100 (48.1)	127 (61.1)	
Stage III	89 (42.8)	36 (17.3)	
Molecular subtype ^*^			< 0.01^c^
Luminal A	38 (18.3)	47 (22.6)	
Luminal B	83 (39.9)	86 (41.3)	
TNBC	37 (17.8)	42 (20.2)	
HER2-enriched	23 (11.1)	26 (12.5)	
Unclassified	27 (13.0)	7 (3.4)	
Estrogen receptor, ER			1.00^2^
Negative	75 (36.1)	75 (36.1)	
Positive	133 (63.9)	133 (63.9)	
Progesterone receptor, PR			0.62^b^
Negative	96 (46.2)	91 (43.8)	
Positive	112 (53.8)	117 (56.2)	
Human epidermal growth factor receptor-2, HER2			
Negative	123 (59.1)	143 (68.8)	
Positive	61 (29.3)	65 (31.2)	
Unknown	24 (11.5)	0 (0.0)	
Primary surgery			0.24^b^
Breast conserving	23 (11.1)	35 (16.8)	
Mastectomy	148 (71.2)	138 (66.3)	
Unknown	37 (17.8)	35 (16.8)	
Chemotherapy ^d^			< 0.01^c^
No	23 (11.1)	9 (4.3)	
Yes	139 (66.8)	186 (89.4)	
Unknown	46 (22.1)	13 (6.2)	
Radiotherapy ^d^			
No	103 (49.5)	97 (46.6)	
Yes	86 (41.3)	111 (53.4)	
Unknown	19 (9.1)	0 (0.0)	
Hormonal therapy ^d^			< 0.01^b^
No	64 (30.8)	80 (38.5)	
Yes	98 (47.1)	115 (55.3)	
Unknown	46 (22.1)	13 (6.2)	

^*^Matching factor. One control per case was randomly selected and individually matched on molecular subtype. If HER2 is unknown for cases, ER and PR were used as matching factors

^a^Linear Model ANOVA

^b^Pearson’s Chi-squared test

^c^Fisher’s Exact Test for Count

^d^information on chemotherapy, radiotherapy, or hormonal therapy in TCGA cohort is not complete

### Somatic mutations

Any mutation in the glucocorticoid pathway was associated with a higher risk of iDFS endpoints (OR 1.66, 95% CI 1.07–2.58; Table 2). A two-fold increase of TMB was associated with 17% elevated odds of iDFS endpoints (95% CI 2–35%). The associations were comparable between the WCH and TCGA samples (P for heterogeneity = 0.76). TMB of the adrenergic pathway and cholinergic pathways was associated with a higher risk of iDFS endpoints for the WCH sample, but not in TCGA. TMB of the dopaminergic or serotoninergic pathway was not associated with iDFS endpoints. The association of any mutation and TMB in the glucocorticoid pathway with iDFS was more pronounced among patients with ER-negative tumors (Table S3).

**Table 2:** The association between tumor mutation burden (TMB) of neuroendocrine pathways and breast cancer prognosis

Pathway	Somatic Mutation ^a^	WCH + TCGA			WCH			TCGA			P for heterogeneity
		Cases	Controls	Odds ratio ^b^	Cases	Controls	Odds ratio	Cases	Controls	Odds ratios	
Adrenergic	No mutation	77	75	Ref	38	49	Ref	39	26	Ref	-
	Any mutation	131	133	1.16 (0.74, 1.81)	58	47	2.01 (1.06, 3.8)	73	86	0.66 (0.34, 1.29)	0.02
	TMB	208	208	1.07 (0.91, 1.24)	96	96	1.28 (1.02, 1.59)	112	112	0.89 (0.7, 1.12)	0.03
Glucocorticoid	No mutation	122	137	Ref	55	65	Ref	67	82	Ref	-
	Any Mutation	86	71	1.66 (1.07, 2.58)	41	31	1.81 (0.95, 3.44)	45	40	1.57 (0.84, 2.95)	0.76
	TMB	208	208	1.17 (1.02, 1.35)	96	96	1.22 (0.99, 1.51)	112	112	1.12 (0.91, 1.38)	0.57
Dopaminergic	No mutation	135	138	Ref	59	68	Ref	76	70	Ref	-
	Any Mutation	73	70	1.07 (0.69, 1.66)	37	28	1.84 (0.95, 3.56)	36	42	0.7 (0.37, 1.33)	0.04
	TMB	208	208	1.02 (0.89, 1.17)	96	96	1.2 (0.98, 1.46)	112	112	0.88 (0.72, 1.08)	0.03
Serotonergic	No mutation	135	140	Ref	60	70	Ref	75	70	Ref	-
	Any Mutation	73	68	1.04 (0.66, 1.62)	36	26	1.74 (0.9, 3.36)	37	42	0.61 (0.32, 1.18)	0.03
	TMB	208	208	1.02 (0.89, 1.18)	96	96	1.2 (0.98, 1.47)	112	112	0.85 (0.68, 1.05)	0.02
Cholinergic	No mutation	61	76	Ref	30	49	Ref	21	27	Ref	-
	Any Mutation	147	132	1.59 (1.01, 2.53)	66	47	2.75 (1.42, 5.32)	81	85	0.87 (0.44, 1.72)	0.02
	TMB	208	208	1.14 (0.97, 1.34)	96	96	1.33 (1.06, 1.67)	112	112	0.95 (0.74, 1.21)	0.05

^a^Somatic mutation burden of candidate genes in each pathway were firstly categorized as mutated or nonmutated, and then treated as a continuous variable (log2 scale)

^b^Odds ratios were estimated using logistic regression models and adjusted for cohort membership, age at diagnosis, menopausal status at diagnosis, molecular subtype, and cancer stage

^c^Between-cohort heterogeneity was calculated using Q-statistics

In additional analyses, we observed largely similar associations with overlapping CIs across models with different adjustments (Table S4). The results remained robust for all pathways after excluding the matching pairs of cases with non-breast-cancer death or patients with neoadjuvant chemotherapy (Table S5). After removing the genes shared between multiple neuroendocrine pathways, the estimates remained similar for the glucocorticoid pathway but not for the cholinergic pathway (Table S5).

### Gene expression

**Fig. 1:**
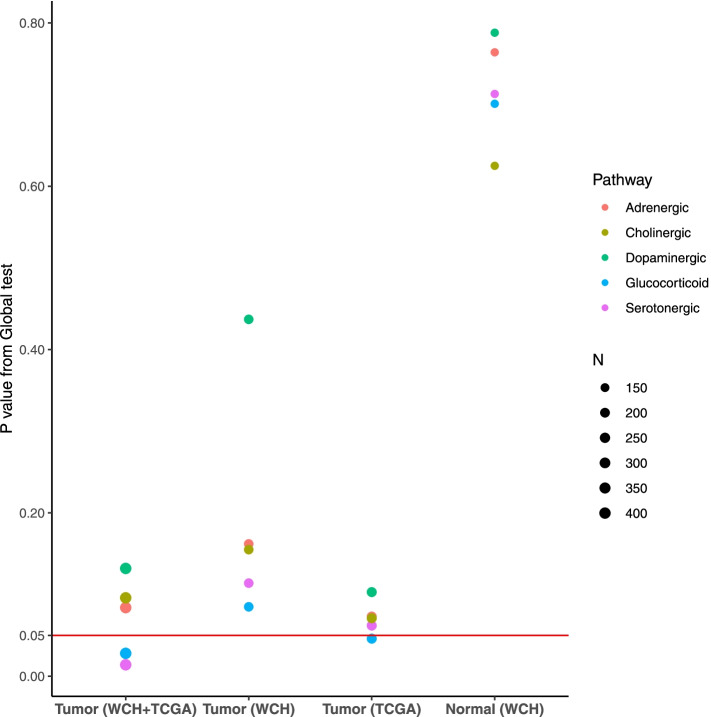
Associations of neuroendocrine pathway gene expression in tumor and matched normal breast tissues with prognosis. *P* value was calculated using Global test, adjusted for cohort membership, age at diagnosis, menopausal status at diagnosis, molecular subtype, and cancer stage

Pathway analysis showed that, in tumor tissue, differential gene expression of the glucocorticoid (*P* = 0.028) and serotonergic (*P* = 0.014) pathways was associated with iDFS (Fig. 1). Although these associations were less significant when separately analyzing the two cohorts, more pronounced results were noted in TCGA which has a larger sample size. Regardless of cohort membership, no association was noted for gene expression of the adrenergic, dopaminergic, or cholinergic pathway in tumor tissue. In contrast, no association between pathways and iDFS was found in normal tissue based on expression data from the WCH sample. In tumor tissue, the associations of all pathways were more pronounced in ER-positive tumors, compared with that in ER-negative tumors, whereas null associations were consistently observed in normal tissue regardless of ER status (Table S6).

Excluding deaths due to causes other than breast cancer or patients with neoadjuvant chemotherapy yielded largely unchanged results for the glucocorticoid and serotonergic pathways (Table S7). The association for the glucocorticoid pathway was somewhat attenuated after removing genes shared between pathways, but the association for the serotonergic pathway remained unchanged.

**Fig. 2:**
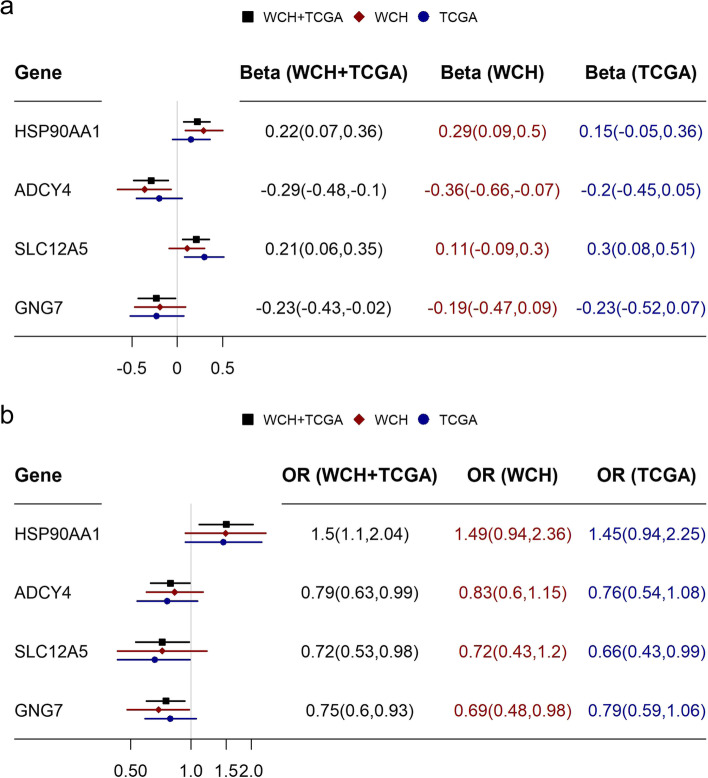
Top genes of the glucocorticoid pathway expressed in tumor tissue associated with mutation and prognosis. a. Association between glucocorticoid pathway tumor mutation burden (TMB) and individual gene expression by linear regression; b. Association between individual gene expression and breast cancer invasive disease-free survival (iDFS) by logistic regression TMB was classified as mutated or not. Gene expression was used as a continuous variable in log2 scale. Both regression models were adjusted for cohort membership, age at diagnosis, menopausal status at diagnosis, molecular subtype, and cancer stage. The genes with *p* values < 0.05 for both analyses were presented in this table

Among the 153 individual genes of the glucocorticoid pathway, we found that gene expression of *HSP90AA1*, *ADCY4*, *SLC12A5*, and *GNG7* was associated with both pathway-level TMB and iDFS (empirical P < 0.05 for both associations; Fig. 2). Expression of these top genes mediated (*HSP90AA1*: 16%, *ADCY4*: 12%, *SLC12A5*: -16%, and *GNG7*: 12%*)* the association between TMB of glucocorticoid pathway and iDFS, although the mediation analysis was underpowered. The associations of the full list of genes of the glucocorticoid pathway are presented in Table S8.

## Discussion

Leveraging omics data from two large clinical cohorts of breast cancer, this study illustrated the contribution of the neuroendocrine pathways to breast cancer prognosis at both the somatic mutation and gene expression levels. We found that a higher somatic mutation burden in the glucocorticoid pathway was associated with an unfavorable iDFS in patients with non-metastatic breast cancer, independent of other known prognostic predictors. Our data further indicated that such association might be mediated through differentially expressed genes of the glucocorticoid pathway in tumor tissue, but not in adjacent normal tissue. We found however limited evidence for the role of other neuroendocrine pathways in breast cancer prognosis.

### Glucocorticoid pathway

Upon activation of the hypothalamic–pituitary–adrenal (HPA) axis, glucocorticoids are synthesized, secreted, and combined to the glucocorticoid receptor and could activate multiple downstream processes involved in breast cancer progression, including cell adhesion, inflammation, chemoresistance, evasion of apoptosis, and angiogenesis [8–11]. Activation of glucocorticoid receptor also regulates the receptor tyrosine kinase ROR1 expression [8] and the Hippo pathway [36], which are importantly involved in cancer progression.

Our data on somatic mutation and gene expression provide further evidence that the glucocorticoid pathway is associated with breast cancer iDFS. A high burden of somatic mutation reflects higher genome instability and chances of altered expression in tumor tissue, whereas no association was found between gene expression in normal breast tissue and iDFS. Moreover, largely similar results were noted in a number of sensitivity analyses, including the exclusion of overlapping genes between the studied neuroendocrine pathways, which argues against the possibility that the association was completely driven by crosstalk between pathways.

We found four top genes in the glucocorticoid pathway which possibly explained the association. These genes have been reported to be associated with cancer survival. Our findings of gene expression and breast cancer prognosis were consistent with the previous evidence for *HSP90AA1* [37], *ADCY4* [38], and *GNG7* [39]. High *SLC12A5* expression was suggested to predict poor survival in patients with bladder urothelial [40], colorectal [41] and ovarian [42] cancers, whereas the association direction in breast cancer was found the opposite in our study. However, our results is consistent between the WCH and TCGA samples as well as in line with public data source (e.g. TCGA data analyzed from cBioportal [43], GEO and EGA data from KM plotter [44]).

We further found that the association between somatic mutation burden of glucocorticoid pathway and iDFS was slightly stronger for ER-negative tumors. However, the association of glucocorticoid pathway signaling was more pronounced in ER-positive tumors. This is partly due to the limited statistical power for ER-negative tumors in pathway analysis which is sensitive to sample size. The transcriptional response of glucocorticoid signaling in breast cancer cells is highly heterogeneous [45]. Glucocorticoids were also suggested to interfere with ER signaling pathway and inhibit ER-mediated cell proliferation [11]. Such regulation in ER-positive tumor may bypass the effect of glucocorticoid pathway mutation on iDFS, while the differential expression at the transcriptome level still results in a different prognosis as noted in pathway analysis.

These results may have important clinical implications. Dexamethasone is commonly used in patients with breast cancer to prevent nausea caused by chemotherapy [46] and was suggested to increase the efficacy of cisplatin [47]. However, dexamethasone may also promote breast cancer metastasis by offsetting the response to paclitaxel [8]. Moreover, abnormal diurnal or nocturnal cortisol rhythm has been associated with disease progression and mortality in breast cancer [48, 49]. More research is needed on the benefits of dexamethasone use in breast cancer patients.

### Adrenergic, dopaminergic, serotonergic, and cholinergic pathways

Our data suggested that TMB of the adrenergic and cholinergic pathways were associated with breast cancer iDFS, although the results were primarily driven by the WCH samples and not shown in the gene expression analysis. The differential expression of the serotonergic pathway in tumor tissue associated with breast cancer iDFS is consistent with findings from a previous report indicating that the poor prognosis of breast cancer was featured by active serotonin production [50]. However, we did not observe an association between the dopaminergic pathway and breast cancer survival, similar to the findings from our earlier studies on prostate cancer [20, 21].

The major strengths of this study include the large cohorts of breast cancer patients with rich information on somatic mutations, gene expression, clinical and follow-up data. The extreme case–control design provides good efficiency and reserves the power from a full cohort [25]. Consistent results from patients with diverse race/ethnical backgrounds in our pooled analysis may help to better generalize the findings across populations. However, several limitations should be noted. First, the two cohorts have heterogeneity. For example, the two cohorts differ in the distributions of race, age, molecular subtype, and length of follow-up. The estimates for glucocorticoid pathway were however consistent between the cohorts, supporting the validity of this finding. Second, only patients with available biospecimens were included in this study. However, the clinical characteristics were largely comparable between patients included in the present analysis and the whole cohort. Third, treatment information was differentially missed between cases and controls in the TCGA sample, resulting in fewer treatment records in cases. This is because cases have a fast-progressing disease and shorter survival and therefore their information is more likely to be incomplete, due to known reasons such as changing health centers and delayed data collection[24]. However, the adjustment for treatment does not change the results substantially in WCH. Another limitation is the multiple tests for five pathways without adjusting for multiplicity. Reassuringly, our claim was restricted to the pathway which showed robust estimates in both cohorts and at both DNA and RNA levels, which diminishes the possibility of a false-positive result. Lastly, the present study aimed to provide biological insights into the link between neuroendocrine pathways and breast cancer prognosis rather than making any claims on functional roles. Future studies are warranted to confirm the biological roles of neuroendocrine pathways in breast cancer prognosis.

## Conclusions

The glucocorticoid pathway may play a role in breast cancer prognosis through differential mutations and expression in tumor tissue. These findings may have implications for the development of novel therapeutic targets for breast cancer, if confirmed in independent investigations.

## Supplementary Information

Below is the link to the electronic supplementary material.

**Additional file 1: Table S1.** List of genes included in the five candidate neuroendocrine pathways. **Table S2.** Clinical characteristics of breast cancer patients from TCGA and WCH cohort, separately. **Table S3.** The associations between tumor mutation burden (TMB) of neuroendocrine pathways and prognosis, stratification analysis by ER status. **Table S4.** The associations between tumor mutation burden (TMB) of neuroendocrine pathways and prognosis with different adjustment. **Table S5.** The associations between tumor mutation burden (TMB) of neuroendocrine pathways and prognosis, in subsets of cohorts or exclusive gene list. **Table S6.** The associations between neuroendocrine pathway gene expression in tumor and normal breast tissue and prognosis, stratification analysis by ER status. **Table S7.** The associations between neuroendocrine pathway gene expression in tumor tissue and prognosis, in subsets of the cohorts or exclusive gene list. **Table S8.** The full list of genes of the glucocorticoid pathway expressed in tumor tissue associated with mutation and prognosis. Supplementary Methods include Matching of cases and controls, Whole exome sequencing and data processing and RNA-sequencing and data processing.

## Data Availability

The Somatic mutation and gene expression data from TCGA were downloaded from GDC portal (https://portal.gdc.cancer.gov). The data from WCH cannot be shared publicly due to the privacy of individuals that participated in the study. The data that support the findings of this study are available on request from the corresponding author.
