# Supplementary material for: Neuroendocrine pathways and breast cancer progression: a pooled analysis of somatic mutations and gene expression from two large breast cancer cohorts
Source: BMC Cancer. 2022 Jun 21;22:680. doi: 10.1186/s12885-022-09779-8 (PMC9210628; doi:10.1186/s12885-022-09779-8)
Supplement: Supplementary file 1 — Additional file 1: Table S1. List of genes included in the five candidate neuroendocrine pathways. Table S2. Clinical characteristics of breast cancer patients from TCGA and WCH cohort, separately. Table S3. The associations between tumor mutation burden (TMB) of neuroendocrine pathways and prognosis, stratification analysis by ER status. Table S4. The associations between tumor mutation burden (TMB) of neuroendocrine pathways and prognosis with different adjustment. Table S5. The associations between tumor mutation burden (TMB) of neuroendocrine pathways and prognosis, in subsets of cohorts or exclusive gene list. Table S6. The associations between neuroendocrine pathway gene expression in tumor and normal breast tissue and prognosis, stratification analysis by ER status. Table S7. The associations between neuroendocrine pathway gene expression in tumor tissue and prognosis, in subsets of the cohorts or exclusive gene list. Table S8. The full list of genes of the glucocorticoid pathway expressed in tumor tissue associated with mutation and prognosis. Supplementary Methods include Matching of cases and controls, Whole exome sequencing and data processing and RNA-sequencing and data processing. [file 12885_2022_9779_MOESM1_ESM.docx]

**Neuroendocrine pathways and breast cancer progression: a pooled analysis of somatic mutations and gene expression from two large breast cancer cohorts**

Kejia Hu­, Chengshi Wang, Chuanxu Luo, Hong Zheng­, Huan Song, Jacob Bergstedt, Katja Fall, Ting Luo, Kamila Czene, Unnur A. Valdimarsdóttir, Fang Fang, Donghao Lu

This file contains supplementary materials of the above article in *BMC Cancer*

**Contents**

[Supplementary tables 3](#_Toc103166747)

[Table S1. List of genes included in the five candidate neuroendocrine pathways 3](#_Toc103166748)

[Table S2. Clinical characteristics of breast cancer patients from TCGA and WCH cohort, separately 26](#_Toc103166749)

[Table S3. The associations between tumor mutation burden (TMB) of neuroendocrine pathways and prognosis, stratification analysis by ER status 27](#_Toc103166750)

[Table S4. The associations between tumor mutation burden (TMB) of neuroendocrine pathways and prognosis with different adjustment 29](#_Toc103166751)

[Table S5. The associations between tumor mutation burden (TMB) of neuroendocrine pathways and prognosis, in subsets of cohorts or exclusive gene list 30](#_Toc103166752)

[Table S6. The associations between neuroendocrine pathway gene expression in tumor and normal breast tissue and prognosis, stratification analysis by ER status 31](#_Toc103166753)

[Table S7. The associations between neuroendocrine pathway gene expression in tumor tissue and prognosis, in subsets of the cohorts or exclusive gene list 31](#_Toc103166754)

[Table S8. The full list of genes of the glucocorticoid pathway expressed in tumor tissue associated with mutation and prognosis 31](#_Toc103166755)

[Supplementary Methods 35](#_Toc103166756)

[Matching of cases and controls 35](#_Toc103166757)

[Whole exome sequencing and data processing 36](#_Toc103166758)

[RNA-sequencing and data processing 39](#_Toc103166759)

# Supplementary tables

# Table S1. List of genes included in the five candidate neuroendocrine pathways

| Pathway | Symbol | Entrez Gene ID | Chromosome | Band | Description |
| --- | --- | --- | --- | --- | --- |
| Adrenergic | *ADAM12* | 8038 | 10 | q26.2 | ADAM metallopeptidase domain 12 |
| Adrenergic | *ADRA1A* | 148 | 8 | p21.2 | adrenoceptor alpha 1A |
| Adrenergic | *ADRA1B* | 147 | 5 | q33.3 | adrenoceptor alpha 1B |
| Adrenergic | *ADRA1D* | 146 | 20 | p13 | adrenoceptor alpha 1D |
| Adrenergic | *ADRA2A* | 150 | 10 | q25.2 | adrenoceptor alpha 2A |
| Adrenergic | *ADRA2B* | 151 | 2 | q11.2 | adrenoceptor alpha 2B |
| Adrenergic | *ADRA2C* | 152 | 4 | p16.3 | adrenoceptor alpha 2C |
| Adrenergic | *ADRB1* | 153 | 10 | q25.3 | adrenoceptor beta 1 |
| Adrenergic | *ADRB2* | 154 | 5 | q32 | adrenoceptor beta 2, surface |
| Adrenergic | *ADRB3* | 155 | 8 | p11.23 | adrenoceptor beta 3 |
| Adrenergic | *GRK2* | 156 | 11 | q13.2 | G protein-coupled receptor kinase 2 |
| Adrenergic | *GRK3* | 157 | 22 | q12.1 | G protein-coupled receptor kinase 3 |
| Adrenergic | *AKT1* | 207 | 14 | q32.33 | v-akt murine thymoma viral oncogene homolog 1 |
| Adrenergic | *AKT2* | 208 | 19 | q13.2 | v-akt murine thymoma viral oncogene homolog 2 |
| Adrenergic | *AKT3* | 10000 | 1 | q44 | v-akt murine thymoma viral oncogene homolog 3 |
| Adrenergic | *ATF2* | 1386 | 2 | q31.1 | activating transcription factor 2 |
| Adrenergic | *ATF3* | 467 | 1 | q32.3 | activating transcription factor 3 |
| Adrenergic | *BAD* | 572 | 11 | q13.1 | BCL2-associated agonist of cell death |
| Adrenergic | *BRAF* | 673 | 7 | q34 | v-raf murine sarcoma viral oncogene homolog B1 |
| Adrenergic | *CAMK2A* | 815 | 5 | q32 | calcium/calmodulin-dependent protein kinase II alpha |
| Adrenergic | *CAMK2B* | 816 | 7 | p13 | calcium/calmodulin-dependent protein kinase II beta |
| Adrenergic | *CAMK2D* | 817 | 4 | q26 | calcium/calmodulin-dependent protein kinase II delta |
| Adrenergic | *CAMK2G* | 818 | 10 | q22.2 | calcium/calmodulin-dependent protein kinase II gamma |
| Adrenergic | *CREB1* | 1385 | 2 | q33.3 | cAMP responsive element binding protein 1 |
| Adrenergic | *CREB3* | 10488 | 9 | p13.3 | cAMP responsive element binding protein 3 |
| Adrenergic | *CREB3L4* | 148327 | 1 | q21.3 | cAMP responsive element binding protein 3-like 4 |
| Adrenergic | *CREB5* | 9586 | 7 | p15.1 | cAMP responsive element binding protein 5 |
| Adrenergic | *CYP2C8* | 1558 | 10 | q23.33 | cytochrome P450, family 2, subfamily C, polypeptide 8 |
| Adrenergic | *EGFR* | 1956 | 7 | p11.2 | epidermal growth factor receptor |
| Adrenergic | *ELK1* | 2002 | X | p11.23 | ELK1, member of ETS oncogene family |
| Adrenergic | *FLT1* | 2321 | 13 | q12.3 | fms-related tyrosine kinase 1 (vascular endothelial growth factor/vascular permeability factor receptor) |
| Adrenergic | *FOS* | 2353 | 14 | q24.3 | FBJ murine osteosarcoma viral oncogene homolog |
| Adrenergic | *FOSB* | 2354 | 19 | q13.32 | FBJ murine osteosarcoma viral oncogene homolog |
| Adrenergic | *GATA1* | 2623 | X | p11.23 | GATA binding protein 1 (globin transcription factor 1) |
| Adrenergic | *GNA11* | 2767 | 19 | p13.3 | guanine nucleotide binding protein (G protein), alpha 11 (Gq class) |
| Adrenergic | *GNA14* | 9630 | 9 | q21.2 | guanine nucleotide binding protein (G protein), alpha 14 |
| Adrenergic | *GNA15* | 2769 | 19 | p13.3 | guanine nucleotide binding protein (G protein), alpha 15 (Gq class) |
| Adrenergic | *GNAI1* | 2770 | 7 | q21.11 | guanine nucleotide binding protein (G protein), alpha inhibiting activity polypeptide 1 |
| Adrenergic | *GNAI2* | 2771 | 3 | p21.31 | guanine nucleotide binding protein (G protein), alpha inhibiting activity polypeptide 2 |
| Adrenergic | *GNAI3* | 2773 | 1 | p13.3 | guanine nucleotide binding protein (G protein), alpha inhibiting activity polypeptide 3 |
| Adrenergic | *GNAQ* | 2776 | 9 | q21.2 | guanine nucleotide binding protein (G protein), q polypeptide |
| Adrenergic | *GNAS* | 2778 | 20 | q13.32 | GNAS complex locus |
| Adrenergic | *GNB1* | 2782 | 1 | p36.33 | guanine nucleotide binding protein (G protein), beta polypeptide 1 |
| Adrenergic | *GNB2* | 2783 | 7 | q22.1 | guanine nucleotide binding protein (G protein), beta polypeptide 2 |
| Adrenergic | *GNB3* | 2784 | 12 | p13.31 | guanine nucleotide binding protein (G protein), beta polypeptide 3 |
| Adrenergic | *GNB4* | 59345 | 3 | q26.33 | guanine nucleotide binding protein (G protein), beta polypeptide 4 |
| Adrenergic | *GNB5* | 10681 | 15 | q21.2 | guanine nucleotide binding protein (G protein), beta 5 |
| Adrenergic | *GNG10* | 2790 | 9 | q31.3 | guanine nucleotide binding protein (G protein), gamma 10 |
| Adrenergic | *GNG11* | 2791 | 7 | q21.3 | guanine nucleotide binding protein (G protein), gamma 11 |
| Adrenergic | *GNG12* | 55970 | 1 | p31.3 | guanine nucleotide binding protein (G protein), gamma 12 |
| Adrenergic | *GNG13* | 51764 | 16 | p13.3 | guanine nucleotide binding protein (G protein), gamma 13 |
| Adrenergic | *GNG2* | 54331 | 14 | q22.1 | guanine nucleotide binding protein (G protein), gamma 2 |
| Adrenergic | *GNG3* | 2785 | 11 | q12.3 | guanine nucleotide binding protein (G protein), gamma 3 |
| Adrenergic | *GNG4* | 2786 | 1 | q42.3 | guanine nucleotide binding protein (G protein), gamma 4 |
| Adrenergic | *GNG5* | 2787 | 1 | p22.3 | guanine nucleotide binding protein (G protein), gamma 5 |
| Adrenergic | *GNG7* | 2788 | 19 | p13.3 | guanine nucleotide binding protein (G protein), gamma 7 |
| Adrenergic | *GNG8* | 94235 | 19 | q13.32 | guanine nucleotide binding protein (G protein), gamma 8 |
| Adrenergic | *GNGT1* | 2792 | 7 | q21.3 | guanine nucleotide binding protein (G protein), gamma transducing activity polypeptide 1 |
| Adrenergic | *GNGT2* | 2793 | 17 | q21.32 | guanine nucleotide binding protein (G protein), gamma transducing activity polypeptide 2 |
| Adrenergic | *GRB2* | 2885 | 17 | q25.1 | growth factor receptor-bound protein 2 |
| Adrenergic | *HBEGF* | 1839 | 5 | q31.3 | heparin-binding EGF-like growth factor |
| Adrenergic | *HRAS* | 3265 | 11 | p15.5 | Harvey rat sarcoma viral oncogene homolog |
| Adrenergic | *ITPR3* | 3710 | 6 | p21.31 | inositol 1,4,5-trisphosphate receptor, type 3 |
| Adrenergic | *JUN* | 3725 | 1 | p32.1 | jun proto-oncogene |
| Adrenergic | *MAP2K1* | 5604 | 15 | q22.31 | mitogen-activated protein kinase kinase 1 |
| Adrenergic | *MAP2K2* | 5605 | 19 | p13.3 | mitogen-activated protein kinase kinase 2 |
| Adrenergic | *MAPK1* | 5594 | 22 | q11.22 | mitogen-activated protein kinase 1 |
| Adrenergic | *MAPK10* | 5602 | 4 | q21.3 | mitogen-activated protein kinase 10 |
| Adrenergic | *MAPK11* | 5600 | 22 | q13.33 | mitogen-activated protein kinase 11 |
| Adrenergic | *MAPK14* | 1432 | 6 | p21.31 | mitogen-activated protein kinase 14 |
| Adrenergic | *MAPK3* | 5595 | 16 | p11.2 | mitogen-activated protein kinase 3 |
| Adrenergic | *MAPK8* | 5599 | 10 | q11.22 | mitogen-activated protein kinase 8 |
| Adrenergic | *MAPK9* | 5601 | 5 | q35.3 | mitogen-activated protein kinase 9 |
| Adrenergic | *NFKB1* | 4790 | 4 | q24 | nuclear factor of kappa light polypeptide gene enhancer in B-cells 1 |
| Adrenergic | *NR3C1* | 2908 | 5 | q31.3 | nuclear receptor subfamily 3, group C, member 1 (glucocorticoid receptor) |
| Adrenergic | *PDPK1* | 5170 | 16 | p13.3 | 3-phosphoinositide dependent protein kinase-1 |
| Adrenergic | *PIK3C2A* | 5286 | 11 | p15.1 | phosphatidylinositol-4-phosphate 3-kinase, catalytic subunit type 2 alpha |
| Adrenergic | *PIK3C2B* | 5287 | 1 | q32.1 | phosphatidylinositol-4-phosphate 3-kinase, catalytic subunit type 2 beta |
| Adrenergic | *PIK3C2G* | 5288 | 12 | p12.3 | phosphatidylinositol-4-phosphate 3-kinase, catalytic subunit type 2 gamma |
| Adrenergic | *PIK3C3* | 5289 | 18 | q12.3 | phosphatidylinositol 3-kinase, catalytic subunit type 3 |
| Adrenergic | *PIK3CA* | 5290 | 3 | q26.32 | phosphatidylinositol-4,5-bisphosphate 3-kinase, catalytic subunit alpha |
| Adrenergic | *PIK3CB* | 5291 | 3 | q22.3 | phosphatidylinositol-4,5-bisphosphate 3-kinase, catalytic subunit beta |
| Adrenergic | *PIK3CD* | 5293 | 1 | p36.22 | phosphatidylinositol-4,5-bisphosphate 3-kinase, catalytic subunit delta |
| Adrenergic | *PIK3CG* | 5294 | 7 | q22.3 | phosphatidylinositol-4,5-bisphosphate 3-kinase, catalytic subunit gamma |
| Adrenergic | *PIK3R1* | 5295 | 5 | q13.1 | phosphoinositide-3-kinase, regulatory subunit 1 (alpha) |
| Adrenergic | *PIK3R2* | 5296 | 19 | p13.11 | phosphoinositide-3-kinase, regulatory subunit 2 (beta) |
| Adrenergic | *PIK3R3* | 8503 | 1 | p34.1 | phosphoinositide-3-kinase, regulatory subunit 3 (gamma) |
| Adrenergic | *PIK3R5* | 23533 | 17 | p13.1 | phosphoinositide-3-kinase, regulatory subunit 5 |
| Adrenergic | *PIK3R6* | 146850 | 17 | p13.1 | phosphoinositide-3-kinase, regulatory subunit 6 |
| Adrenergic | *PLA2G4A* | 5321 | 1 | q31.1 | phospholipase A2, group IVA (cytosolic, calcium-dependent) |
| Adrenergic | *PLA2G4C* | 8605 | 19 | q13.33 | phospholipase A2, group IVC (cytosolic, calcium-independent) |
| Adrenergic | *PLA2G6* | 8398 | 22 | q13.1 | phospholipase A2, group VI (cytosolic, calcium-independent) |
| Adrenergic | *PLCB1* | 23236 | 20 | p12.3 | phospholipase C, beta 1 (phosphoinositide-specific) |
| Adrenergic | *PLCB2* | 5330 | 15 | q15.1 | phospholipase C, beta 2 |
| Adrenergic | *PLCB3* | 5331 | 11 | q13.1 | phospholipase C, beta 3 (phosphatidylinositol-specific) |
| Adrenergic | *PLCD1* | 5333 | 3 | p22.2 | phospholipase C, delta 1 |
| Adrenergic | *PLCE1* | 51196 | 10 | q23.33 | phospholipase C, epsilon 1 |
| Adrenergic | *PPP2CA* | 5515 | 5 | q31.1 | protein phosphatase 2, catalytic subunit, alpha isozyme |
| Adrenergic | *PPP2CB* | 5516 | 8 | p12 | protein phosphatase 2, catalytic subunit, beta isozyme |
| Adrenergic | *PPP2R1A* | 5518 | 19 | q13.41 | protein phosphatase 2, regulatory subunit A, alpha |
| Adrenergic | *PPP2R2A* | 5520 | 8 | p21.2 | protein phosphatase 2, regulatory subunit B, alpha |
| Adrenergic | *PPP2R2B* | 5521 | 5 | q32 | protein phosphatase 2, regulatory subunit B, beta |
| Adrenergic | *PPP2R3B* | 28227 | X | p22.33 | protein phosphatase 2, regulatory subunit B'', beta |
| Adrenergic | *PPP2R5A* | 5525 | 1 | q32.3 | protein phosphatase 2, regulatory subunit B', alpha |
| Adrenergic | *PPP2R5B* | 5526 | 11 | q13.1 | protein phosphatase 2, regulatory subunit B', beta |
| Adrenergic | *PPP2R5D* | 5528 | 6 | p21.1 | protein phosphatase 2, regulatory subunit B', delta |
| Adrenergic | *PPP2R5E* | 5529 | 14 | q23.2 | protein phosphatase 2, regulatory subunit B', epsilon isoform |
| Adrenergic | *PRKACA* | 5566 | 19 | p13.12 | protein kinase, cAMP-dependent, catalytic, alpha |
| Adrenergic | *PRKACB* | 5567 | 1 | p31.1 | protein kinase, cAMP-dependent, catalytic, beta |
| Adrenergic | *PRKACG* | 5568 | 9 | q21.11 | protein kinase, cAMP-dependent, catalytic, gamma |
| Adrenergic | *PRKAR1A* | 5573 | 17 | q24.2 | protein kinase, cAMP-dependent, regulatory, type I, alpha |
| Adrenergic | *PRKAR2A* | 5576 | 3 | p21.31 | protein kinase, cAMP-dependent, regulatory, type II, alpha |
| Adrenergic | *PTK2* | 5747 | 8 | q24.3 | PTK2 protein tyrosine kinase 2 |
| Adrenergic | *PTK2B* | 2185 | 8 | p21.2 | PTK2B protein tyrosine kinase 2 beta |
| Adrenergic | *RAF1* | 5894 | 3 | p25.2 | v-raf-1 murine leukemia viral oncogene homolog 1 |
| Adrenergic | *RAP1A* | 5906 | 1 | p13.2 | RAP1A, member of RAS oncogene family |
| Adrenergic | *RAP2B* | 5912 | 3 | q25.2 | RAP2B, member of RAS oncogene family |
| Adrenergic | *RAPGEF2* | 9693 | 4 | q32.1 | Rap guanine nucleotide exchange factor (GEF) 2 |
| Adrenergic | *RAPGEF3* | 10411 | 12 | q13.11 | Rap guanine nucleotide exchange factor (GEF) 3 |
| Adrenergic | *RASGRP1* | 10125 | 15 | q14 | RAS guanyl releasing protein 1 (calcium and DAG-regulated) |
| Adrenergic | *REL* | 5966 | 2 | p16.1 | v-rel reticuloendotheliosis viral oncogene homolog (avian) |
| Adrenergic | *RELA* | 5970 | 11 | q13.1 | v-rel reticuloendotheliosis viral oncogene homolog A (avian) |
| Adrenergic | *SHC1* | 6464 | 1 | q21.3 | SHC (Src homology 2 domain containing) transforming protein 1 |
| Adrenergic | *SOS1* | 6654 | 2 | p22.1 | son of sevenless homolog 1 (Drosophila) |
| Adrenergic | *SOS2* | 6655 | 14 | q21.3 | son of sevenless homolog 2 (Drosophila) |
| Adrenergic | *SPRY1* | 10252 | 4 | q28.1 | sprouty homolog 1, antagonist of FGF signaling (Drosophila) |
| Adrenergic | *SRC* | 6714 | 20 | q11.23 | v-src sarcoma (Schmidt-Ruppin A-2) viral oncogene homolog (avian) |
| Adrenergic | *STAT3* | 6774 | 17 | q21.2 | signal transducer and activator of transcription 3 (acute-phase response factor) |
| Adrenergic | *TGM2* | 7052 | 20 | q11.23 | transglutaminase 2 |
| Adrenergic | *VEGFA* | 7422 | 6 | p21.1 | vascular endothelial growth factor A |
| Adrenergic | *VEGFB* | 7423 | 11 | q13.1 | vascular endothelial growth factor B |
| Adrenergic | *VEGFC* | 7424 | 4 | q34.3 | vascular endothelial growth factor C |
| Adrenergic | *ADCY1* | 107 | 7 | p12.3 | adenylate cyclase 1 |
| Adrenergic | *ADCY2* | 108 | 5 | p15.31 | adenylate cyclase 2 |
| Adrenergic | *ADCY3* | 109 | 2 | p23.3 | adenylate cyclase 3 |
| Adrenergic | *ADCY4* | 196883 | 14 | q12 | adenylate cyclase 4 |
| Adrenergic | *ADCY5* | 111 | 3 | q21.1 | adenylate cyclase 5 |
| Adrenergic | *ADCY6* | 112 | 12 | q13.12 | adenylate cyclase 6 |
| Adrenergic | *ADCY7* | 113 | 16 | q12.1 | adenylate cyclase 7 |
| Adrenergic | *ADCY8* | 114 | 8 | q24.22 | adenylate cyclase 8 |
| Adrenergic | *ADCY9* | 115 | 16 | p13.3 | adenylate cyclase 9 |
| Adrenergic | *ATP1A1* | 476 | 1 | p13.1 | ATPase Na+/K+ transporting subunit alpha 1 |
| Adrenergic | *ATP1A2* | 477 | 1 | q23.2 | ATPase Na+/K+ transporting subunit alpha 2 |
| Adrenergic | *ATP1A3* | 478 | 19 | q13.2 | ATPase Na+/K+ transporting subunit alpha 3 |
| Adrenergic | *ATP1A4* | 480 | 1 | q23.2 | ATPase Na+/K+ transporting subunit alpha 4 |
| Adrenergic | *ATP1B4* | 23439 | X | q24 | ATPase Na+/K+ transporting family member beta 4 |
| Adrenergic | *ATP1B1* | 481 | 1 | q24.2 | ATPase Na+/K+ transporting subunit beta 1 |
| Adrenergic | *ATP1B2* | 482 | 17 | p13.1 | ATPase Na+/K+ transporting subunit beta 2 |
| Adrenergic | *ATP1B3* | 483 | 3 | q23 | ATPase Na+/K+ transporting subunit beta 3 |
| Adrenergic | *FXYD2* | 486 | 11 | q23.3 | FXYD domain containing ion transport regulator 2 |
| Adrenergic | *SLC9A1* | 6548 | 1 | p36.11 | solute carrier family 9 member A1 |
| Adrenergic | *KCNQ1* | 3784 | 11 | p15.5 | potassium voltage-gated channel subfamily Q member 1 |
| Adrenergic | *KCNE1* | 3753 | 21 | q22.12 | potassium voltage-gated channel subfamily E regulatory subunit 1 |
| Adrenergic | *KCNE1B* | 1.03E+08 | 21 | p11.2 | potassium voltage-gated channel subfamily E regulatory subunit 1B |
| Adrenergic | *ATP2B1* | 490 | 12 | q21.33 | ATPase plasma membrane Ca2+ transporting 1 |
| Adrenergic | *ATP2B3* | 492 | X | q28 | ATPase plasma membrane Ca2+ transporting 3 |
| Adrenergic | *ATP2B4* | 493 | 1 | q32.1 | ATPase plasma membrane Ca2+ transporting 4 |
| Adrenergic | *ATP2B2* | 491 | 3 | p25.3 | ATPase plasma membrane Ca2+ transporting 2 |
| Adrenergic | *SLC8A1* | 6546 | 2 | p22.1 | solute carrier family 8 member A1 |
| Adrenergic | *SLC8A2* | 6543 | 19 | q13.32 | solute carrier family 8 member A2 |
| Adrenergic | *SLC8A3* | 6547 | 14 | q24.2 | solute carrier family 8 member A3 |
| Adrenergic | *SCN5A* | 6331 | 3 | p22.2 | sodium voltage-gated channel alpha subunit 5 |
| Adrenergic | *SCN7A* | 6332 | 2 | q24.3 | sodium voltage-gated channel alpha subunit 7 |
| Adrenergic | *SCN1B* | 6324 | 19 | q13.11 | sodium voltage-gated channel beta subunit 1 |
| Adrenergic | *SCN4B* | 6330 | 11 | q23.3 | sodium voltage-gated channel beta subunit 4 |
| Adrenergic | *CACNA1C* | 775 | 12 | p13.33 | calcium voltage-gated channel subunit alpha1 C |
| Adrenergic | *CACNA1D* | 776 | 3 | p21.1 | calcium voltage-gated channel subunit alpha1 D |
| Adrenergic | *CACNA1F* | 778 | X | p11.23 | calcium voltage-gated channel subunit alpha1 F |
| Adrenergic | *CACNA1S* | 779 | 1 | q32.1 | calcium voltage-gated channel subunit alpha1 S |
| Adrenergic | *CACNB1* | 782 | 17 | q12 | calcium voltage-gated channel auxiliary subunit beta 1 |
| Adrenergic | *CACNB2* | 783 | 10 | p12.33 | calcium voltage-gated channel auxiliary subunit beta 2 |
| Adrenergic | *CACNB3* | 784 | 12 | q13.12 | calcium voltage-gated channel auxiliary subunit beta 3 |
| Adrenergic | *CACNB4* | 785 | 2 | q23.3 | calcium voltage-gated channel auxiliary subunit beta 4 |
| Adrenergic | *CACNA2D1* | 781 | 7 | q21.11 | calcium voltage-gated channel auxiliary subunit alpha2delta 1 |
| Adrenergic | *CACNA2D2* | 9254 | 3 | p21.31 | calcium voltage-gated channel auxiliary subunit alpha2delta 2 |
| Adrenergic | *CACNA2D3* | 55799 | 3 | p21.1 | calcium voltage-gated channel auxiliary subunit alpha2delta 3 |
| Adrenergic | *CACNA2D4* | 93589 | 12 | p13.33 | calcium voltage-gated channel auxiliary subunit alpha2delta 4 |
| Adrenergic | *CACNG1* | 786 | 17 | q24.2 | calcium voltage-gated channel auxiliary subunit gamma 1 |
| Adrenergic | *CACNG2* | 10369 | 22 | q12.3 | calcium voltage-gated channel auxiliary subunit gamma 2 |
| Adrenergic | *CACNG3* | 10368 | 16 | p12.1 | calcium voltage-gated channel auxiliary subunit gamma 3 |
| Adrenergic | *CACNG4* | 27092 | 17 | q24.2 | calcium voltage-gated channel auxiliary subunit gamma 4 |
| Adrenergic | *CACNG5* | 27091 | 17 | q24.2 | calcium voltage-gated channel auxiliary subunit gamma 5 |
| Adrenergic | *CACNG6* | 59285 | 19 | q13.42 | calcium voltage-gated channel auxiliary subunit gamma 6 |
| Adrenergic | *CACNG7* | 59284 | 19 | q13.42 | calcium voltage-gated channel auxiliary subunit gamma 7 |
| Adrenergic | *CACNG8* | 59283 | 19 | q13.42 | calcium voltage-gated channel auxiliary subunit gamma 8 |
| Adrenergic | *CALML3* | 810 | 10 | p15.1 | calmodulin like 3 |
| Adrenergic | *CALM2* | 805 | 2 | p21 | calmodulin 2 |
| Adrenergic | *CALM3* | 808 | 19 | q13.32 | calmodulin 3 |
| Adrenergic | *CALM1* | 801 | 14 | q32.11 | calmodulin 1 |
| Adrenergic | *CALML6* | 163688 | 1 | p36.33 | calmodulin like 6 |
| Adrenergic | *CALML5* | 51806 | 10 | p15.1 | calmodulin like 5 |
| Adrenergic | *CALML4* | 91860 | 15 | q23 | calmodulin like 4 |
| Adrenergic | *RYR2* | 6262 | 1 | q43 | ryanodine receptor 2 |
| Adrenergic | *PPP2R1B* | 5519 | 11 | q23.1 | protein phosphatase 2 scaffold subunit Abeta |
| Adrenergic | *PPP2R2C* | 5522 | 4 | p16.1 | protein phosphatase 2 regulatory subunit Bgamma |
| Adrenergic | *PPP2R2D* | 55844 | 10 | q26.3 | protein phosphatase 2 regulatory subunit Bdelta |
| Adrenergic | *PPP2R3C* | 55012 | 14 | q13.2 | protein phosphatase 2 regulatory subunit B''gamma |
| Adrenergic | *PPP2R3A* | 5523 | 3 | q22.2 | protein phosphatase 2 regulatory subunit B''alpha |
| Adrenergic | *PPP2R5C* | 5527 | 14 | q32.31 | protein phosphatase 2 regulatory subunit B'gamma |
| Adrenergic | *PPP1CA* | 5499 | 11 | q13.2 | protein phosphatase 1 catalytic subunit alpha |
| Adrenergic | *PPP1CB* | 5500 | 2 | p23.2 | protein phosphatase 1 catalytic subunit beta |
| Adrenergic | *PPP1CC* | 5501 | 12 | q24.11 | protein phosphatase 1 catalytic subunit gamma |
| Adrenergic | *TNNC1* | 7134 | 3 | p21.1 | troponin C1, slow skeletal and cardiac type |
| Adrenergic | *TNNI3* | 7137 | 19 | q13.42 | troponin I3, cardiac type |
| Adrenergic | *TNNT2* | 7139 | 1 | q32.1 | troponin T2, cardiac type |
| Adrenergic | *TPM1* | 7168 | 15 | q22.2 | tropomyosin 1 |
| Adrenergic | *TPM2* | 7169 | 9 | p13.3 | tropomyosin 2 |
| Adrenergic | *TPM3* | 7170 | 1 | q21.3 | tropomyosin 3 |
| Adrenergic | *TPM4* | 7171 | 19 | p13.12 | tropomyosin 4 |
| Adrenergic | *ACTC1* | 70 | 15 | q14 | actin alpha cardiac muscle 1 |
| Adrenergic | *MYH7* | 4625 | 14 | q11.2 | myosin heavy chain 7 |
| Adrenergic | *MYH6* | 4624 | 14 | q11.2 | myosin heavy chain 6 |
| Adrenergic | *MYL2* | 4633 | 12 | q24.11 | myosin light chain 2 |
| Adrenergic | *MYL3* | 4634 | 3 | p21.31 | myosin light chain 3 |
| Adrenergic | *MYL4* | 4635 | 17 | q21.32 | myosin light chain 4 |
| Adrenergic | *PLN* | 5350 | 6 | q22.31 | phospholamban |
| Adrenergic | *ATP2A1* | 487 | 16 | p11.2 | ATPase sarcoplasmic/endoplasmic reticulum Ca2+ transporting 1 |
| Adrenergic | *ATP2A3* | 489 | 17 | p13.2 | ATPase sarcoplasmic/endoplasmic reticulum Ca2+ transporting 3 |
| Adrenergic | *ATP2A2* | 488 | 12 | q24.11 | ATPase sarcoplasmic/endoplasmic reticulum Ca2+ transporting 2 |
| Adrenergic | *AGT* | 183 | 1 | q42.2 | angiotensinogen |
| Adrenergic | *AGTR1* | 185 | 3 | q24 | angiotensin II receptor type 1 |
| Adrenergic | *AGTR2* | 186 | X | q23 | angiotensin II receptor type 2 |
| Adrenergic | *PLCB4* | 5332 | 20 | p12.3 | phospholipase C beta 4 |
| Adrenergic | *PRKCA* | 5578 | 17 | q24.2 | protein kinase C alpha |
| Adrenergic | *PPP1R1A* | 5502 | 12 | q13.2 | protein phosphatase 1 regulatory inhibitor subunit 1A |
| Adrenergic | *RPS6KA5* | 9252 | 14 | q32.11 | ribosomal protein S6 kinase A5 |
| Adrenergic | *ATF4* | 468 | 22 | q13.1 | activating transcription factor 4 |
| Adrenergic | *CREB3L1* | 90993 | 11 | p11.2 | cAMP responsive element binding protein 3 like 1 |
| Adrenergic | *CREB3L2* | 64764 | 7 | q33 | cAMP responsive element binding protein 3 like 2 |
| Adrenergic | *CREB3L3* | 84699 | 19 | p13.3 | cAMP responsive element binding protein 3 like 3 |
| Adrenergic | *ATF6B* | 1388 | 6 | p21.32 | activating transcription factor 6 beta |
| Adrenergic | *CREM* | 1390 | 10 | p11.21 | cAMP responsive element modulator |
| Adrenergic | *BCL2* | 596 | 18 | q21.33 | BCL2 apoptosis regulator |
| Adrenergic | *RAPGEF4* | 11069 | 2 | q31.1 | Rap guanine nucleotide exchange factor 4 |
| Adrenergic | *MAPK12* | 6300 | 22 | q13.33 | mitogen-activated protein kinase 12 |
| Adrenergic | *MAPK13* | 5603 | 6 | p21.31 | mitogen-activated protein kinase 13 |
| Glucocorticoid | *CDKN1A* | 1026 | 6 | p21.2 | cyclin-dependent kinase inhibitor 1A (p21, Cip1) |
| Glucocorticoid | *CEBPA* | 1050 | 19 | q13.11 | CCAAT/enhancer binding protein (C/EBP), alpha |
| Glucocorticoid | *CEBPB* | 1051 | 20 | q13.13 | CCAAT/enhancer binding protein (C/EBP), beta |
| Glucocorticoid | *CREB1* | 1385 | 2 | q33.3 | cAMP responsive element binding protein 1 |
| Glucocorticoid | *CREB3* | 10488 | 9 | p13.3 | cAMP responsive element binding protein 3 |
| Glucocorticoid | *CREB3L4* | 148327 | 1 | q21.3 | cAMP responsive element binding protein 3-like 4 |
| Glucocorticoid | *CREB5* | 9586 | 7 | p15.1 | cAMP responsive element binding protein 5 |
| Glucocorticoid | *CREBBP* | 1387 | 16 | p13.3 | CREB binding protein |
| Glucocorticoid | *ELK1* | 2002 | X | p11.23 | ELK1, member of ETS oncogene family |
| Glucocorticoid | *FKBP4* | 2288 | 12 | p13.33 | FK506 binding protein 4, 59kDa |
| Glucocorticoid | *FOS* | 2353 | 14 | q24.3 | FBJ murine osteosarcoma viral oncogene homolog |
| Glucocorticoid | *FOXO3* | 2309 | 6 | q21 | forkhead box O3 |
| Glucocorticoid | *GLCCI1* | 113263 | 7 | p21.3 | glucocorticoid induced transcript 1 |
| Glucocorticoid | *GMEB1* | 10691 | 1 | p35.3 | glucocorticoid modulatory element binding protein 1 |
| Glucocorticoid | *GMEB2* | 26205 | 20 | q13.33 | glucocorticoid modulatory element binding protein 2 |
| Glucocorticoid | *GTF2A1* | 2957 | 14 | q31.1 | general transcription factor IIA, 1, 19/37kDa |
| Glucocorticoid | *GTF2A2* | 2958 | 15 | q22.2 | general transcription factor IIA, 2, 12kDa |
| Glucocorticoid | *GTF2B* | 2959 | 1 | p22.2 | general transcription factor IIB |
| Glucocorticoid | *GTF2E1* | 2960 | 3 | q13.33 | general transcription factor IIE, polypeptide 1, alpha 56kDa |
| Glucocorticoid | *GTF2E2* | 2961 | 8 | p12 | general transcription factor IIE, polypeptide 2, beta 34kDa |
| Glucocorticoid | *GTF2F1* | 2962 | 19 | p13.3 | general transcription factor IIF, polypeptide 1, 74kDa |
| Glucocorticoid | *GTF2F2* | 2963 | 13 | q14.12 | general transcription factor IIF, polypeptide 2, 30kDa |
| Glucocorticoid | *GTF2H1* | 2965 | 11 | p15.1 | general transcription factor IIH, polypeptide 1, 62kDa |
| Glucocorticoid | *GTF2H2* | 2966 | 5 | q13.2 | general transcription factor IIH, polypeptide 2, 44kDa |
| Glucocorticoid | *GTF2H3* | 2967 | 12 | q24.31 | general transcription factor IIH, polypeptide 3, 34kDa |
| Glucocorticoid | *GTF2H4* | 2968 | 6 | p21.33 | general transcription factor IIH, polypeptide 4, 52kDa |
| Glucocorticoid | *GTF2H5* | 404672 | 6 | q25.3 | general transcription factor IIH, polypeptide 5 |
| Glucocorticoid | *HSP90AA1* | 3320 | 14 | q32.31 | heat shock protein 90kDa alpha (cytosolic), class A member 1 |
| Glucocorticoid | *HSP90AB1* | 3326 | 6 | p21.1 | heat shock protein 90kDa alpha (cytosolic), class B member 1 |
| Glucocorticoid | *HSPA1A* | 3303 | 6 | p21.33 | heat shock 70kDa protein 1A |
| Glucocorticoid | *HSPA1B* | 3304 | 6 | p21.33 | heat shock 70kDa protein 1B |
| Glucocorticoid | *HSPA4* | 3308 | 5 | q31.1 | heat shock 70kDa protein 4 |
| Glucocorticoid | *JUN* | 3725 | 1 | p32.1 | jun proto-oncogene |
| Glucocorticoid | *JUNB* | 3726 | 19 | p13.13 | jun B proto-oncogene |
| Glucocorticoid | *JUND* | 3727 | 19 | p13.11 | jun D proto-oncogene |
| Glucocorticoid | *KAT2B* | 8850 | 3 | p24.3 | K(lysine) acetyltransferase 2B |
| Glucocorticoid | *MAPK1* | 5594 | 22 | q11.22 | mitogen-activated protein kinase 1 |
| Glucocorticoid | *MAPK11* | 5600 | 22 | q13.33 | mitogen-activated protein kinase 11 |
| Glucocorticoid | *MAPK12* | 6300 | 22 | q13.33 | mitogen-activated protein kinase 12 |
| Glucocorticoid | *MAPK13* | 5603 | 6 | p21.31 | mitogen-activated protein kinase 13 |
| Glucocorticoid | *MAPK14* | 1432 | 6 | p21.31 | mitogen-activated protein kinase 14 |
| Glucocorticoid | *MAPK8* | 5599 | 10 | q11.22 | mitogen-activated protein kinase 8 |
| Glucocorticoid | *MMP13* | 4322 | 11 | q22.2 | matrix metallopeptidase 13 (collagenase 3) |
| Glucocorticoid | *NCOA1* | 8648 | 2 | p23.3 | nuclear receptor coactivator 1 |
| Glucocorticoid | *NCOA2* | 10499 | 8 | q13.3 | nuclear receptor coactivator 2 |
| Glucocorticoid | *NFKB1* | 4790 | 4 | q24 | nuclear factor of kappa light polypeptide gene enhancer in B-cells 1 |
| Glucocorticoid | *NFKBIA* | 4792 | 14 | q13.2 | nuclear factor of kappa light polypeptide gene enhancer in B-cells inhibitor, alpha |
| Glucocorticoid | *NR3C1* | 2908 | 5 | q31.3 | nuclear receptor subfamily 3, group C, member 1 (glucocorticoid receptor) |
| Glucocorticoid | *POU2F1* | 5451 | 1 | q24.2 | POU class 2 homeobox 1 |
| Glucocorticoid | *POU2F2* | 5452 | 19 | q13.2 | POU class 2 homeobox 2 |
| Glucocorticoid | *PTGES3* | 10728 | 12 | q13.3 | prostaglandin E synthase 3 (cytosolic) |
| Glucocorticoid | *SERPINE1* | 5054 | 7 | q22.1 | serpin peptidase inhibitor, clade E (nexin, plasminogen activator inhibitor type 1), member 1 |
| Glucocorticoid | *SGK1* | 6446 | 6 | q23.2 | serum/glucocorticoid regulated kinase 1 |
| Glucocorticoid | *SLC22A1* | 6580 | 6 | q25.3 | solute carrier family 22 (organic cation transporter), member 1 |
| Glucocorticoid | *SLC22A2* | 6582 | 6 | q25.3 | solute carrier family 22 (organic cation transporter), member 2 |
| Glucocorticoid | *SMAD3* | 4088 | 15 | q22.33 | SMAD family member 3 |
| Glucocorticoid | *SMAD4* | 4089 | 18 | q21.2 | SMAD family member 4 |
| Glucocorticoid | *SRC* | 6714 | 20 | q11.23 | v-src sarcoma (Schmidt-Ruppin A-2) viral oncogene homolog (avian) |
| Glucocorticoid | *STAT5A* | 6776 | 17 | q21.2 | signal transducer and activator of transcription 5A |
| Glucocorticoid | *STAT5B* | 6777 | 17 | q21.2 | signal transducer and activator of transcription 5B |
| Glucocorticoid | *SUMO1* | 7341 | 2 | q33.1 | small ubiquitin-like modifier 1 |
| Glucocorticoid | *TBP* | 6908 | 6 | q27 | TATA box binding protein |
| Glucocorticoid | *TGFB1* | 7040 | 19 | q13.2 | transforming growth factor, beta 1 |
| Glucocorticoid | *UBE2I* | 7329 | 16 | p13.3 | ubiquitin-conjugating enzyme E2I |
| Glucocorticoid | *XIAP* | 331 | X | q25 | X-linked inhibitor of apoptosis |
| Glucocorticoid | *SLC38A1* | 81539 | 12 | q13.11 | solute carrier family 38 member 1 |
| Glucocorticoid | *SLC38A2* | 54407 | 12 | q13.11 | solute carrier family 38 member 2 |
| Glucocorticoid | *GLS2* | 27165 | 12 | q13.3 | glutaminase 2 |
| Glucocorticoid | *GLS* | 2744 | 2 | q32.2 | glutaminase |
| Glucocorticoid | *GAD1* | 2571 | 2 | q31.1 | glutamate decarboxylase 1 |
| Glucocorticoid | *GAD2* | 2572 | 10 | p12.1 | glutamate decarboxylase 2 |
| Glucocorticoid | *SLC32A1* | 140679 | 20 | q11.23 | solute carrier family 32 member 1 |
| Glucocorticoid | *ABAT* | 18 | 16 | p13.2 | 4-aminobutyrate aminotransferase |
| Glucocorticoid | *GABRA1* | 2554 | 5 | q34 | gamma-aminobutyric acid type A receptor subunit alpha1 |
| Glucocorticoid | *GABRA2* | 2555 | 4 | p12 | gamma-aminobutyric acid type A receptor subunit alpha2 |
| Glucocorticoid | *GABRA3* | 2556 | X | q28 | gamma-aminobutyric acid type A receptor subunit alpha3 |
| Glucocorticoid | *GABRA4* | 2557 | 4 | p12 | gamma-aminobutyric acid type A receptor subunit alpha4 |
| Glucocorticoid | *GABRA5* | 2558 | 15 | q12 | gamma-aminobutyric acid type A receptor subunit alpha5 |
| Glucocorticoid | *GABRA6* | 2559 | 5 | q34 | gamma-aminobutyric acid type A receptor subunit alpha6 |
| Glucocorticoid | *GABRB1* | 2560 | 4 | p12 | gamma-aminobutyric acid type A receptor subunit beta1 |
| Glucocorticoid | *GABRB3* | 2562 | 15 | q12 | gamma-aminobutyric acid type A receptor subunit beta3 |
| Glucocorticoid | *GABRB2* | 2561 | 5 | q34 | gamma-aminobutyric acid type A receptor subunit beta2 |
| Glucocorticoid | *GABRG1* | 2565 | 4 | p12 | gamma-aminobutyric acid type A receptor subunit gamma1 |
| Glucocorticoid | *GABRG2* | 2566 | 5 | q34 | gamma-aminobutyric acid type A receptor subunit gamma2 |
| Glucocorticoid | *GABRG3* | 2567 | 15 | q12 | gamma-aminobutyric acid type A receptor subunit gamma3 |
| Glucocorticoid | *GABRD* | 2563 | 1 | p36.33 | gamma-aminobutyric acid type A receptor subunit delta |
| Glucocorticoid | *GABRE* | 2564 | X | q28 | gamma-aminobutyric acid type A receptor subunit epsilon |
| Glucocorticoid | *GABRQ* | 55879 | X | q28 | gamma-aminobutyric acid type A receptor subunit theta |
| Glucocorticoid | *GABRP* | 2568 | 5 | q35.1 | gamma-aminobutyric acid type A receptor subunit pi |
| Glucocorticoid | *PRKACA* | 5566 | 19 | p13.12 | protein kinase cAMP-activated catalytic subunit alpha |
| Glucocorticoid | *PRKACB* | 5567 | 1 | p31.1 | protein kinase cAMP-activated catalytic subunit beta |
| Glucocorticoid | *PRKACG* | 5568 | 9 | q21.11 | protein kinase cAMP-activated catalytic subunit gamma |
| Glucocorticoid | *PRKCA* | 5578 | 17 | q24.2 | protein kinase C alpha |
| Glucocorticoid | *PRKCB* | 5579 | 16 | p12.2 | protein kinase C beta |
| Glucocorticoid | *PRKCG* | 5582 | 19 | q13.42 | protein kinase C gamma |
| Glucocorticoid | *HAP1* | 9001 | 17 | q21.2 | huntingtin associated protein 1 |
| Glucocorticoid | *GABARAP* | 11337 | 17 | p13.1 | GABA type A receptor-associated protein |
| Glucocorticoid | *GABARAPL1* | 23710 | 12 | p13.2 | GABA type A receptor associated protein like 1 |
| Glucocorticoid | *GABARAPL2* | 11345 | 16 | q23.1 | GABA type A receptor associated protein like 2 |
| Glucocorticoid | *NSF* | 4905 | 17 | q21.31 | N-ethylmaleimide sensitive factor, vesicle fusing ATPase |
| Glucocorticoid | *TRAK2* | 66008 | 2 | q33.1 | trafficking kinesin protein 2 |
| Glucocorticoid | *PLCL1* | 5334 | 2 | q33.1 | phospholipase C like 1 (inactive) |
| Glucocorticoid | *GPHN* | 10243 | 14 | q23.3 | gephyrin |
| Glucocorticoid | *SLC12A5* | 57468 | 20 | q13.12 | solute carrier family 12 member 5 |
| Glucocorticoid | *GABRR1* | 2569 | 6 | q15 | gamma-aminobutyric acid type A receptor subunit rho1 |
| Glucocorticoid | *GABRR2* | 2570 | 6 | q15 | gamma-aminobutyric acid type A receptor subunit rho2 |
| Glucocorticoid | *GABRR3* | 200959 | 3 | q11.2 | gamma-aminobutyric acid type A receptor subunit rho3 |
| Glucocorticoid | *CACNA1A* | 773 | 19 | p13.13 | calcium voltage-gated channel subunit alpha1 A |
| Glucocorticoid | *CACNA1B* | 774 | 9 | q34.3 | calcium voltage-gated channel subunit alpha1 B |
| Glucocorticoid | *CACNA1C* | 775 | 12 | p13.33 | calcium voltage-gated channel subunit alpha1 C |
| Glucocorticoid | *CACNA1D* | 776 | 3 | p21.1 | calcium voltage-gated channel subunit alpha1 D |
| Glucocorticoid | *CACNA1F* | 778 | X | p11.23 | calcium voltage-gated channel subunit alpha1 F |
| Glucocorticoid | *CACNA1S* | 779 | 1 | q32.1 | calcium voltage-gated channel subunit alpha1 S |
| Glucocorticoid | *GABBR1* | 2550 | 6 | p22.1 | gamma-aminobutyric acid type B receptor subunit 1 |
| Glucocorticoid | *GABBR2* | 9568 | 9 | q22.33 | gamma-aminobutyric acid type B receptor subunit 2 |
| Glucocorticoid | *GNAI1* | 2770 | 7 | q21.11 | G protein subunit alpha i1 |
| Glucocorticoid | *GNAI3* | 2773 | 1 | p13.3 | G protein subunit alpha i3 |
| Glucocorticoid | *GNAI2* | 2771 | 3 | p21.31 | G protein subunit alpha i2 |
| Glucocorticoid | *GNAO1* | 2775 | 16 | q13 | G protein subunit alpha o1 |
| Glucocorticoid | *GNB1* | 2782 | 1 | p36.33 | G protein subunit beta 1 |
| Glucocorticoid | *GNB2* | 2783 | 7 | q22.1 | G protein subunit beta 2 |
| Glucocorticoid | *GNB3* | 2784 | 12 | p13.31 | G protein subunit beta 3 |
| Glucocorticoid | *GNB4* | 59345 | 3 | q26.33 | G protein subunit beta 4 |
| Glucocorticoid | *GNB5* | 10681 | 15 | q21.2 | G protein subunit beta 5 |
| Glucocorticoid | *GNG2* | 54331 | 14 | q22.1 | G protein subunit gamma 2 |
| Glucocorticoid | *GNG3* | 2785 | 11 | q12.3 | G protein subunit gamma 3 |
| Glucocorticoid | *GNG4* | 2786 | 1 | q42.3 | G protein subunit gamma 4 |
| Glucocorticoid | *GNG5* | 2787 | 1 | p22.3 | G protein subunit gamma 5 |
| Glucocorticoid | *GNG7* | 2788 | 19 | p13.3 | G protein subunit gamma 7 |
| Glucocorticoid | *GNG8* | 94235 | 19 | q13.32 | G protein subunit gamma 8 |
| Glucocorticoid | *GNG10* | 2790 | 9 | q31.3 | G protein subunit gamma 10 |
| Glucocorticoid | *GNG11* | 2791 | 7 | q21.3 | G protein subunit gamma 11 |
| Glucocorticoid | *GNG12* | 55970 | 1 | p31.3 | G protein subunit gamma 12 |
| Glucocorticoid | *GNG13* | 51764 | 16 | p13.3 | G protein subunit gamma 13 |
| Glucocorticoid | *GNGT1* | 2792 | 7 | q21.3 | G protein subunit gamma transducin 1 |
| Glucocorticoid | *GNGT2* | 2793 | 17 | q21.32 | G protein subunit gamma transducin 2 |
| Glucocorticoid | *ADCY1* | 107 | 7 | p12.3 | adenylate cyclase 1 |
| Glucocorticoid | *ADCY2* | 108 | 5 | p15.31 | adenylate cyclase 2 |
| Glucocorticoid | *ADCY3* | 109 | 2 | p23.3 | adenylate cyclase 3 |
| Glucocorticoid | *ADCY4* | 196883 | 14 | q12 | adenylate cyclase 4 |
| Glucocorticoid | *ADCY5* | 111 | 3 | q21.1 | adenylate cyclase 5 |
| Glucocorticoid | *ADCY6* | 112 | 12 | q13.12 | adenylate cyclase 6 |
| Glucocorticoid | *ADCY7* | 113 | 16 | q12.1 | adenylate cyclase 7 |
| Glucocorticoid | *ADCY8* | 114 | 8 | q24.22 | adenylate cyclase 8 |
| Glucocorticoid | *ADCY9* | 115 | 16 | p13.3 | adenylate cyclase 9 |
| Glucocorticoid | *KCNJ6* | 3763 | 21 | q22.13 | potassium inwardly rectifying channel subfamily J member 6 |
| Glucocorticoid | *SLC6A1* | 6529 | 3 | p25.3 | solute carrier family 6 member 1 |
| Glucocorticoid | *SLC6A13* | 6540 | 12 | p13.33 | solute carrier family 6 member 13 |
| Glucocorticoid | *SLC6A11* | 6538 | 3 | p25.3 | solute carrier family 6 member 11 |
| Glucocorticoid | *SLC6A12* | 6539 | 12 | p13.33 | solute carrier family 6 member 12 |
| Glucocorticoid | *GLUL* | 2752 | 1 | q25.3 | glutamate-ammonia ligase |
| Glucocorticoid | *SLC38A3* | 10991 | 3 | p21.31 | solute carrier family 38 member 3 |
| Glucocorticoid | *SLC38A5* | 92745 | X | p11.23 | solute carrier family 38 member 5 |
| Dopaminergic | *BCL2L1* | 598 | 20 | q11.21 | BCL2-like 1 |
| Dopaminergic | *CALY* | 50632 | 10 | q26.3 | calcyon neuron-specific vesicular protein |
| Dopaminergic | *DRD1* | 1812 | 5 | q35.2 | dopamine receptor D1 |
| Dopaminergic | *DRD2* | 1813 | 11 | q23.2 | dopamine receptor D2 |
| Dopaminergic | *DRD3* | 1814 | 3 | q13.31 | dopamine receptor D3 |
| Dopaminergic | *DRD4* | 1815 | 11 | p15.5 | dopamine receptor D4 |
| Dopaminergic | *DRD5* | 1816 | 4 | p16.1 | dopamine receptor D5 |
| Dopaminergic | *FOS* | 2353 | 14 | q24.3 | FBJ murine osteosarcoma viral oncogene homolog |
| Dopaminergic | *GNAL* | 2774 | 18 | p11.21 | guanine nucleotide binding protein (G protein), alpha activating activity polypeptide, olfactory type |
| Dopaminergic | *GNAQ* | 2776 | 9 | q21.2 | guanine nucleotide binding protein (G protein), q polypeptide |
| Dopaminergic | *GNAS* | 2778 | 20 | q13.32 | GNAS complex locus |
| Dopaminergic | *ITPR1* | 3708 | 3 | p26.1 | inositol 1,4,5-trisphosphate receptor, type 1 |
| Dopaminergic | *ITPR2* | 3709 | 12 | p11.23 | inositol 1,4,5-trisphosphate receptor, type 2 |
| Dopaminergic | *ITPR3* | 3710 | 6 | p21.31 | inositol 1,4,5-trisphosphate receptor, type 3 |
| Dopaminergic | *MAP2K1* | 5604 | 15 | q22.31 | mitogen-activated protein kinase kinase 1 |
| Dopaminergic | *MAPK1* | 5594 | 22 | q11.22 | mitogen-activated protein kinase 1 |
| Dopaminergic | *MAPK10* | 5602 | 4 | q21.3 | mitogen-activated protein kinase 10 |
| Dopaminergic | *MAPK11* | 5600 | 22 | q13.33 | mitogen-activated protein kinase 11 |
| Dopaminergic | *MAPK12* | 6300 | 22 | q13.33 | mitogen-activated protein kinase 12 |
| Dopaminergic | *MAPK13* | 5603 | 6 | p21.31 | mitogen-activated protein kinase 13 |
| Dopaminergic | *MAPK14* | 1432 | 6 | p21.31 | mitogen-activated protein kinase 14 |
| Dopaminergic | *MAPK8* | 5599 | 10 | q11.22 | mitogen-activated protein kinase 8 |
| Dopaminergic | *MAPK9* | 5601 | 5 | q35.3 | mitogen-activated protein kinase 9 |
| Dopaminergic | *PLCB1* | 23236 | 20 | p12.3 | phospholipase C, beta 1 (phosphoinositide-specific) |
| Dopaminergic | *PLCB2* | 5330 | 15 | q15.1 | phospholipase C, beta 2 |
| Dopaminergic | *PLCB3* | 5331 | 11 | q13.1 | phospholipase C, beta 3 (phosphatidylinositol-specific) |
| Dopaminergic | *PLCB4* | 5332 | 20 | p12.3 | phospholipase C, beta 4 |
| Dopaminergic | *PPP1R1B* | 84152 | 17 | q12 | protein phosphatase 1, regulatory (inhibitor) subunit 1B |
| Dopaminergic | *PRKACA* | 5566 | 19 | p13.12 | protein kinase, cAMP-dependent, catalytic, alpha |
| Dopaminergic | *PRKACB* | 5567 | 1 | p31.1 | protein kinase, cAMP-dependent, catalytic, beta |
| Dopaminergic | *PRKACG* | 5568 | 9 | q21.11 | protein kinase, cAMP-dependent, catalytic, gamma |
| Dopaminergic | *PRKCA* | 5578 | 17 | q24.2 | protein kinase C, alpha |
| Dopaminergic | *PRKCB* | 5579 | 16 | p12.2 | protein kinase C, beta |
| Dopaminergic | *PRKCG* | 5582 | 19 | q13.42 | protein kinase C, gamma |
| Dopaminergic | *PRKX* | 5613 | X | p22.33 | protein kinase, X-linked |
| Dopaminergic | *SCN1A* | 6323 | 2 | q24.3 | sodium channel, voltage-gated, type I, alpha subunit |
| Dopaminergic | *SRC* | 6714 | 20 | q11.23 | v-src sarcoma (Schmidt-Ruppin A-2) viral oncogene homolog (avian) |
| Dopaminergic | *STAT3* | 6774 | 17 | q21.2 | signal transducer and activator of transcription 3 (acute-phase response factor) |
| Dopaminergic | *TH* | 7054 | 11 | p15.5 | tyrosine hydroxylase |
| Dopaminergic | *DDC* | 1644 | 7 | p12.1 | dopa decarboxylase |
| Dopaminergic | *SLC18A1* | 6570 | 8 | p21.3 | solute carrier family 18 member A1 |
| Dopaminergic | *SLC18A2* | 6571 | 10 | q25.3 | solute carrier family 18 member A2 |
| Dopaminergic | *CALML3* | 810 | 10 | p15.1 | calmodulin like 3 |
| Dopaminergic | *CALM2* | 805 | 2 | p21 | calmodulin 2 |
| Dopaminergic | *CALM3* | 808 | 19 | q13.32 | calmodulin 3 |
| Dopaminergic | *CALM1* | 801 | 14 | q32.11 | calmodulin 1 |
| Dopaminergic | *CALML6* | 163688 | 1 | p36.33 | calmodulin like 6 |
| Dopaminergic | *CALML5* | 51806 | 10 | p15.1 | calmodulin like 5 |
| Dopaminergic | *CALML4* | 91860 | 15 | q23 | calmodulin like 4 |
| Dopaminergic | *CAMK2A* | 815 | 5 | q32 | calcium/calmodulin dependent protein kinase II alpha |
| Dopaminergic | *CAMK2D* | 817 | 4 | q26 | calcium/calmodulin dependent protein kinase II delta |
| Dopaminergic | *CAMK2B* | 816 | 7 | p13 | calcium/calmodulin dependent protein kinase II beta |
| Dopaminergic | *CAMK2G* | 818 | 10 | q22.2 | calcium/calmodulin dependent protein kinase II gamma |
| Dopaminergic | *PPP3CA* | 5530 | 4 | q24 | protein phosphatase 3 catalytic subunit alpha |
| Dopaminergic | *PPP3CB* | 5532 | 10 | q22.2 | protein phosphatase 3 catalytic subunit beta |
| Dopaminergic | *PPP3CC* | 5533 | 8 | p21.3 | protein phosphatase 3 catalytic subunit gamma |
| Dopaminergic | *ADCY5* | 111 | 3 | q21.1 | adenylate cyclase 5 |
| Dopaminergic | *CREB1* | 1385 | 2 | q33.3 | cAMP responsive element binding protein 1 |
| Dopaminergic | *ATF2* | 1386 | 2 | q31.1 | activating transcription factor 2 |
| Dopaminergic | *ATF4* | 468 | 22 | q13.1 | activating transcription factor 4 |
| Dopaminergic | *CREB3* | 10488 | 9 | p13.3 | cAMP responsive element binding protein 3 |
| Dopaminergic | *CREB3L1* | 90993 | 11 | p11.2 | cAMP responsive element binding protein 3 like 1 |
| Dopaminergic | *CREB3L2* | 64764 | 7 | q33 | cAMP responsive element binding protein 3 like 2 |
| Dopaminergic | *CREB3L3* | 84699 | 19 | p13.3 | cAMP responsive element binding protein 3 like 3 |
| Dopaminergic | *CREB3L4* | 148327 | 1 | q21.3 | cAMP responsive element binding protein 3 like 4 |
| Dopaminergic | *CREB5* | 9586 | 7 | p15.1 | cAMP responsive element binding protein 5 |
| Dopaminergic | *ATF6B* | 1388 | 6 | p21.32 | activating transcription factor 6 beta |
| Dopaminergic | *PPP1CA* | 5499 | 11 | q13.2 | protein phosphatase 1 catalytic subunit alpha |
| Dopaminergic | *PPP1CB* | 5500 | 2 | p23.2 | protein phosphatase 1 catalytic subunit beta |
| Dopaminergic | *PPP1CC* | 5501 | 12 | q24.11 | protein phosphatase 1 catalytic subunit gamma |
| Dopaminergic | *CACNA1C* | 775 | 12 | p13.33 | calcium voltage-gated channel subunit alpha1 C |
| Dopaminergic | *CACNA1D* | 776 | 3 | p21.1 | calcium voltage-gated channel subunit alpha1 D |
| Dopaminergic | *CACNA1A* | 773 | 19 | p13.13 | calcium voltage-gated channel subunit alpha1 A |
| Dopaminergic | *CACNA1B* | 774 | 9 | q34.3 | calcium voltage-gated channel subunit alpha1 B |
| Dopaminergic | *KCNJ3* | 3760 | 2 | q24.1 | potassium inwardly rectifying channel subfamily J member 3 |
| Dopaminergic | *KCNJ6* | 3763 | 21 | q22.13 | potassium inwardly rectifying channel subfamily J member 6 |
| Dopaminergic | *KCNJ9* | 3765 | 1 | q23.2 | potassium inwardly rectifying channel subfamily J member 9 |
| Dopaminergic | *KCNJ5* | 3762 | 11 | q24.3 | potassium inwardly rectifying channel subfamily J member 5 |
| Dopaminergic | *GNAI1* | 2770 | 7 | q21.11 | G protein subunit alpha i1 |
| Dopaminergic | *GNAI3* | 2773 | 1 | p13.3 | G protein subunit alpha i3 |
| Dopaminergic | *GNAI2* | 2771 | 3 | p21.31 | G protein subunit alpha i2 |
| Dopaminergic | *GNAO1* | 2775 | 16 | q13 | G protein subunit alpha o1 |
| Dopaminergic | *GNB1* | 2782 | 1 | p36.33 | G protein subunit beta 1 |
| Dopaminergic | *GNB2* | 2783 | 7 | q22.1 | G protein subunit beta 2 |
| Dopaminergic | *GNB3* | 2784 | 12 | p13.31 | G protein subunit beta 3 |
| Dopaminergic | *GNB4* | 59345 | 3 | q26.33 | G protein subunit beta 4 |
| Dopaminergic | *GNB5* | 10681 | 15 | q21.2 | G protein subunit beta 5 |
| Dopaminergic | *GNG2* | 54331 | 14 | q22.1 | G protein subunit gamma 2 |
| Dopaminergic | *GNG3* | 2785 | 11 | q12.3 | G protein subunit gamma 3 |
| Dopaminergic | *GNG4* | 2786 | 1 | q42.3 | G protein subunit gamma 4 |
| Dopaminergic | *GNG5* | 2787 | 1 | p22.3 | G protein subunit gamma 5 |
| Dopaminergic | *GNG7* | 2788 | 19 | p13.3 | G protein subunit gamma 7 |
| Dopaminergic | *GNG8* | 94235 | 19 | q13.32 | G protein subunit gamma 8 |
| Dopaminergic | *GNG10* | 2790 | 9 | q31.3 | G protein subunit gamma 10 |
| Dopaminergic | *GNG11* | 2791 | 7 | q21.3 | G protein subunit gamma 11 |
| Dopaminergic | *GNG12* | 55970 | 1 | p31.3 | G protein subunit gamma 12 |
| Dopaminergic | *GNG13* | 51764 | 16 | p13.3 | G protein subunit gamma 13 |
| Dopaminergic | *GNGT1* | 2792 | 7 | q21.3 | G protein subunit gamma transducin 1 |
| Dopaminergic | *GNGT2* | 2793 | 17 | q21.32 | G protein subunit gamma transducin 2 |
| Dopaminergic | *ARRB1* | 408 | 11 | q13.4 | arrestin beta 1 |
| Dopaminergic | *ARRB2* | 409 | 17 | p13.2 | arrestin beta 2 |
| Dopaminergic | *PPP2CA* | 5515 | 5 | q31.1 | protein phosphatase 2 catalytic subunit alpha |
| Dopaminergic | *PPP2CB* | 5516 | 8 | p12 | protein phosphatase 2 catalytic subunit beta |
| Dopaminergic | *PPP2R1B* | 5519 | 11 | q23.1 | protein phosphatase 2 scaffold subunit Abeta |
| Dopaminergic | *PPP2R1A* | 5518 | 19 | q13.41 | protein phosphatase 2 scaffold subunit Aalpha |
| Dopaminergic | *PPP2R2A* | 5520 | 8 | p21.2 | protein phosphatase 2 regulatory subunit Balpha |
| Dopaminergic | *PPP2R2B* | 5521 | 5 | q32 | protein phosphatase 2 regulatory subunit Bbeta |
| Dopaminergic | *PPP2R2C* | 5522 | 4 | p16.1 | protein phosphatase 2 regulatory subunit Bgamma |
| Dopaminergic | *PPP2R2D* | 55844 | 10 | q26.3 | protein phosphatase 2 regulatory subunit Bdelta |
| Dopaminergic | *PPP2R3B* | 28227 | X | p22.33 | protein phosphatase 2 regulatory subunit B''beta |
| Dopaminergic | *PPP2R3C* | 55012 | 14 | q13.2 | protein phosphatase 2 regulatory subunit B''gamma |
| Dopaminergic | *PPP2R3A* | 5523 | 3 | q22.2 | protein phosphatase 2 regulatory subunit B''alpha |
| Dopaminergic | *PPP2R5B* | 5526 | 11 | q13.1 | protein phosphatase 2 regulatory subunit B'beta |
| Dopaminergic | *PPP2R5C* | 5527 | 14 | q32.31 | protein phosphatase 2 regulatory subunit B'gamma |
| Dopaminergic | *PPP2R5D* | 5528 | 6 | p21.1 | protein phosphatase 2 regulatory subunit B'delta |
| Dopaminergic | *PPP2R5E* | 5529 | 14 | q23.2 | protein phosphatase 2 regulatory subunit B'epsilon |
| Dopaminergic | *PPP2R5A* | 5525 | 1 | q32.3 | protein phosphatase 2 regulatory subunit B'alpha |
| Dopaminergic | *AKT1* | 207 | 14 | q32.33 | AKT serine/threonine kinase 1 |
| Dopaminergic | *AKT2* | 208 | 19 | q13.2 | AKT serine/threonine kinase 2 |
| Dopaminergic | *AKT3* | 10000 | 1 | q44 | AKT serine/threonine kinase 3 |
| Dopaminergic | *GSK3A* | 2931 | 19 | q13.2 | glycogen synthase kinase 3 alpha |
| Dopaminergic | *GSK3B* | 2932 | 3 | q13.33 | glycogen synthase kinase 3 beta |
| Dopaminergic | *GRIN2A* | 2903 | 16 | p13.2 | glutamate ionotropic receptor NMDA type subunit 2A |
| Dopaminergic | *GRIN2B* | 2904 | 12 | p13.1 | glutamate ionotropic receptor NMDA type subunit 2B |
| Dopaminergic | *GRIA1* | 2890 | 5 | q33.2 | glutamate ionotropic receptor AMPA type subunit 1 |
| Dopaminergic | *GRIA2* | 2891 | 4 | q32.1 | glutamate ionotropic receptor AMPA type subunit 2 |
| Dopaminergic | *GRIA3* | 2892 | X | q25 | glutamate ionotropic receptor AMPA type subunit 3 |
| Dopaminergic | *GRIA4* | 2893 | 11 | q22.3 | glutamate ionotropic receptor AMPA type subunit 4 |
| Dopaminergic | *KIF5A* | 3798 | 12 | q13.3 | kinesin family member 5A |
| Dopaminergic | *KIF5B* | 3799 | 10 | p11.22 | kinesin family member 5B |
| Dopaminergic | *KIF5C* | 3800 | 2 | q23.1 | kinesin family member 5C |
| Dopaminergic | *CLOCK* | 9575 | 4 | q12 | clock circadian regulator |
| Dopaminergic | *ARNTL* | 406 | 11 | p15.3 | aryl hydrocarbon receptor nuclear translocator like |
| Dopaminergic | *SLC6A3* | 6531 | 5 | p15.33 | solute carrier family 6 member 3 |
| Dopaminergic | *MAOB* | 4129 | X | p11.3 | monoamine oxidase B |
| Dopaminergic | *MAOA* | 4128 | X | p11.3 | monoamine oxidase A |
| Dopaminergic | *COMT* | 1312 | 22 | q11.21 | catechol-O-methyltransferase |
| Dopaminergic | *LRTOMT* | 220074 | 11 | q13.4 | leucine rich transmembrane and O-methyltransferase domain containing |
| Serotonergic | *ARAF* | 369 | X | p11.3 | v-raf murine sarcoma 3611 viral oncogene homolog |
| Serotonergic | *BRAF* | 673 | 7 | q34 | v-raf murine sarcoma viral oncogene homolog B |
| Serotonergic | *CACNA1A* | 773 | 19 | p13.13 | calcium channel, voltage-dependent, P/Q type, alpha 1A subunit |
| Serotonergic | *CACNA1B* | 774 | 9 | q34.3 | calcium channel, voltage-dependent, N type, alpha 1B subunit |
| Serotonergic | *CACNA1D* | 776 | 3 | p21.1 | calcium channel, voltage-dependent, L type, alpha 1D subunit |
| Serotonergic | *CACNA1F* | 778 | X | p11.23 | calcium channel, voltage-dependent, L type, alpha 1F subunit |
| Serotonergic | *CACNA1S* | 779 | 1 | q32.1 | calcium channel, voltage-dependent, L type, alpha 1S subunit |
| Serotonergic | *DUSP1* | 1843 | 5 | q35.1 | dual specificity phosphatase 1 |
| Serotonergic | *EIF4EBP1* | 1978 | 8 | p11.23 | eukaryotic translation initiation factor 4E binding protein 1 |
| Serotonergic | *FOXO3* | 2309 | 6 | q21 | forkhead box O3 |
| Serotonergic | *GNAI1* | 2770 | 7 | q21.11 | guanine nucleotide binding protein (G protein), alpha inhibiting activity polypeptide 1 |
| Serotonergic | *GNAI2* | 2771 | 3 | p21.31 | guanine nucleotide binding protein (G protein), alpha inhibiting activity polypeptide 2 |
| Serotonergic | *GNAI3* | 2773 | 1 | p13.3 | guanine nucleotide binding protein (G protein), alpha inhibiting activity polypeptide 3 |
| Serotonergic | *GNAO1* | 2775 | 16 | q13 | guanine nucleotide binding protein (G protein), alpha activating activity polypeptide O |
| Serotonergic | *GNAQ* | 2776 | 9 | q21.2 | guanine nucleotide binding protein (G protein), q polypeptide |
| Serotonergic | *GNB1* | 2782 | 1 | p36.33 | guanine nucleotide binding protein (G protein), beta polypeptide 1 |
| Serotonergic | *GNB2* | 2783 | 7 | q22.1 | guanine nucleotide binding protein (G protein), beta polypeptide 2 |
| Serotonergic | *GNB3* | 2784 | 12 | p13.31 | guanine nucleotide binding protein (G protein), beta polypeptide 3 |
| Serotonergic | *GNB4* | 59345 | 3 | q26.33 | guanine nucleotide binding protein (G protein), beta polypeptide 4 |
| Serotonergic | *GNB5* | 10681 | 15 | q21.2 | guanine nucleotide binding protein (G protein), beta 5 |
| Serotonergic | *GNG10* | 2790 | 9 | q31.3 | guanine nucleotide binding protein (G protein), gamma 10 |
| Serotonergic | *GNG11* | 2791 | 7 | q21.3 | guanine nucleotide binding protein (G protein), gamma 11 |
| Serotonergic | *GNG12* | 55970 | 1 | p31.3 | guanine nucleotide binding protein (G protein), gamma 12 |
| Serotonergic | *GNG13* | 51764 | 16 | p13.3 | guanine nucleotide binding protein (G protein), gamma 13 |
| Serotonergic | *GNG2* | 54331 | 14 | q22.1 | guanine nucleotide binding protein (G protein), gamma 2 |
| Serotonergic | *GNG3* | 2785 | 11 | q12.3 | guanine nucleotide binding protein (G protein), gamma 3 |
| Serotonergic | *GNG4* | 2786 | 1 | q42.3 | guanine nucleotide binding protein (G protein), gamma 4 |
| Serotonergic | *GNG5* | 2787 | 1 | p22.3 | guanine nucleotide binding protein (G protein), gamma 5 |
| Serotonergic | *GNG7* | 2788 | 19 | p13.3 | guanine nucleotide binding protein (G protein), gamma 7 |
| Serotonergic | *GNG8* | 94235 | 19 | q13.32 | guanine nucleotide binding protein (G protein), gamma 8 |
| Serotonergic | *GNGT1* | 2792 | 7 | q21.3 | guanine nucleotide binding protein (G protein), gamma transducing activity polypeptide 1 |
| Serotonergic | *GNGT2* | 2793 | 17 | q21.32 | guanine nucleotide binding protein (G protein), gamma transducing activity polypeptide 2 |
| Serotonergic | *HRAS* | 3265 | 11 | p15.5 | Harvey rat sarcoma viral oncogene homolog |
| Serotonergic | *HTR1A* | 3350 | 5 | q12.3 | 5-hydroxytryptamine (serotonin) receptor 1A, G protein-coupled |
| Serotonergic | *HTR1B* | 3351 | 6 | q14.1 | 5-hydroxytryptamine (serotonin) receptor 1B, G protein-coupled |
| Serotonergic | *HTR1D* | 3352 | 1 | p36.12 | 5-hydroxytryptamine (serotonin) receptor 1D, G protein-coupled |
| Serotonergic | *HTR1E* | 3354 | 6 | q14.3 | 5-hydroxytryptamine (serotonin) receptor 1E, G protein-coupled |
| Serotonergic | *HTR1F* | 3355 | 3 | p11.2 | 5-hydroxytryptamine (serotonin) receptor 1F, G protein-coupled |
| Serotonergic | *HTR2A* | 3356 | 13 | q14.2 | 5-hydroxytryptamine (serotonin) receptor 2A, G protein-coupled |
| Serotonergic | *HTR2B* | 3357 | 2 | q37.1 | 5-hydroxytryptamine (serotonin) receptor 2B, G protein-coupled |
| Serotonergic | *HTR2C* | 3358 | X | q23 | 5-hydroxytryptamine (serotonin) receptor 2C, G protein-coupled |
| Serotonergic | *HTR3A* | 3359 | 11 | q23.2 | 5-hydroxytryptamine (serotonin) receptor 3A, ionotropic |
| Serotonergic | *HTR3B* | 9177 | 11 | q23.2 | 5-hydroxytryptamine (serotonin) receptor 3B, ionotropic |
| Serotonergic | *HTR3C* | 170572 | 3 | q27.1 | 5-hydroxytryptamine (serotonin) receptor 3C, ionotropic |
| Serotonergic | *HTR3D* | 200909 | 3 | q27.1 | 5-hydroxytryptamine (serotonin) receptor 3D, ionotropic |
| Serotonergic | *HTR3E* | 285242 | 3 | q27.1 | 5-hydroxytryptamine (serotonin) receptor 3E, ionotropic |
| Serotonergic | *HTR4* | 3360 | 5 | q32 | 5-hydroxytryptamine (serotonin) receptor 4, G protein-coupled |
| Serotonergic | *HTR5A* | 3361 | 7 | q36.2 | 5-hydroxytryptamine (serotonin) receptor 5A, G protein-coupled |
| Serotonergic | *HTR6* | 3362 | 1 | p36.13 | 5-hydroxytryptamine (serotonin) receptor 6, G protein-coupled |
| Serotonergic | *HTR7* | 3363 | 10 | q23.31 | 5-hydroxytryptamine (serotonin) receptor 7, adenylate cyclase-coupled |
| Serotonergic | *ITPR1* | 3708 | 3 | p26.1 | inositol 1,4,5-trisphosphate receptor, type 1 |
| Serotonergic | *ITPR2* | 3709 | 12 | p11.23 | inositol 1,4,5-trisphosphate receptor, type 2 |
| Serotonergic | *ITPR3* | 3710 | 6 | p21.31 | inositol 1,4,5-trisphosphate receptor, type 3 |
| Serotonergic | *JAK2* | 3717 | 9 | p24.1 | Janus kinase 2 |
| Serotonergic | *KRAS* | 3845 | 12 | p12.1 | Kirsten rat sarcoma viral oncogene homolog |
| Serotonergic | *KYNU* | 8942 | 2 | q22.2 | kynureninase |
| Serotonergic | *GNG14* | 1.05E+08 | 19 | p13.13 | guanine nucleotide binding protein (G protein), gamma 12-like |
| Serotonergic | *MAP2K1* | 5604 | 15 | q22.31 | mitogen-activated protein kinase kinase 1 |
| Serotonergic | *MAP2K2* | 5605 | 19 | p13.3 | mitogen-activated protein kinase kinase 2 |
| Serotonergic | *MAPK1* | 5594 | 22 | q11.22 | mitogen-activated protein kinase 1 |
| Serotonergic | *MAPK3* | 5595 | 16 | p11.2 | mitogen-activated protein kinase 3 |
| Serotonergic | *MTOR* | 2475 | 1 | p36.22 | mechanistic target of rapamycin (serine/threonine kinase) |
| Serotonergic | *NRAS* | 4893 | 1 | p13.2 | neuroblastoma RAS viral (v-ras) oncogene homolog |
| Serotonergic | *PLCB1* | 23236 | 20 | p12.3 | phospholipase C, beta 1 (phosphoinositide-specific) |
| Serotonergic | *PLCB2* | 5330 | 15 | q15.1 | phospholipase C, beta 2 |
| Serotonergic | *PLCB3* | 5331 | 11 | q13.1 | phospholipase C, beta 3 (phosphatidylinositol-specific) |
| Serotonergic | *PLCB4* | 5332 | 20 | p12.3 | phospholipase C, beta 4 |
| Serotonergic | *PRKCA* | 5578 | 17 | q24.2 | protein kinase C, alpha |
| Serotonergic | *PRKCB* | 5579 | 16 | p12.2 | protein kinase C, beta |
| Serotonergic | *PRKCG* | 5582 | 19 | q13.42 | protein kinase C, gamma |
| Serotonergic | *RAF1* | 5894 | 3 | p25.2 | v-raf-1 murine leukemia viral oncogene homolog 1 |
| Serotonergic | *RPS6KB1* | 6198 | 17 | q23.1 | ribosomal protein S6 kinase, 70kDa, polypeptide 1 |
| Serotonergic | *SRC* | 6714 | 20 | q11.23 | v-src sarcoma (Schmidt-Ruppin A-2) viral oncogene homolog (avian) |
| Serotonergic | *STAT3* | 6774 | 17 | q21.2 | signal transducer and activator of transcription 3 (acute-phase response factor) |
| Serotonergic | *STAT5B* | 6777 | 17 | q21.2 | signal transducer and activator of transcription 5B |
| Serotonergic | *TPH2* | 121278 | 12 | q21.1 | tryptophan hydroxylase 2 |
| Serotonergic | *TPH1* | 7166 | 11 | p15.1 | tryptophan hydroxylase 1 |
| Serotonergic | *DDC* | 1644 | 7 | p12.1 | dopa decarboxylase |
| Serotonergic | *SLC18A1* | 6570 | 8 | p21.3 | solute carrier family 18 member A1 |
| Serotonergic | *SLC18A2* | 6571 | 10 | q25.3 | solute carrier family 18 member A2 |
| Serotonergic | *CACNA1C* | 775 | 12 | p13.33 | calcium voltage-gated channel subunit alpha1 C |
| Serotonergic | *PLA2G4E* | 123745 | 15 | q15.1 | phospholipase A2 group IVE |
| Serotonergic | *PLA2G4A* | 5321 | 1 | q31.1 | phospholipase A2 group IVA |
| Serotonergic | *JMJD7_PLA2G4B* | 8681 | 15 | q15.1 | JMJD7-PLA2G4B readthrough |
| Serotonergic | *PLA2G4B* | 1E+08 | 15 | q15.1 | phospholipase A2 group IVB |
| Serotonergic | *PLA2G4C* | 8605 | 19 | q13.33 | phospholipase A2 group IVC |
| Serotonergic | *PLA2G4D* | 283748 | 15 | q15.1 | phospholipase A2 group IVD |
| Serotonergic | *PLA2G4F* | 255189 | 15 | q15.1 | phospholipase A2 group IVF |
| Serotonergic | *CYP2C8* | 1558 | 10 | q23.33 | cytochrome P450 family 2 subfamily C member 8 |
| Serotonergic | *CYP2C9* | 1559 | 10 | q23.33 | cytochrome P450 family 2 subfamily C member 9 |
| Serotonergic | *CYP2C18* | 1562 | 10 | q23.33 | cytochrome P450 family 2 subfamily C member 18 |
| Serotonergic | *CYP2C19* | 1557 | 10 | q23.33 | cytochrome P450 family 2 subfamily C member 19 |
| Serotonergic | *CYP2D6* | 1565 | 22 | q13.2 | cytochrome P450 family 2 subfamily D member 6 |
| Serotonergic | *LOC107987478* | #N/A | #N/A | #N/A | cytochrome P450 2D6-like |
| Serotonergic | *CYP2D7* | #N/A | #N/A | #N/A | cytochrome P450 family 2 subfamily D member 7 (gene/pseudogene) |
| Serotonergic | *CYP2J2* | 1573 | 1 | p32.1 | cytochrome P450 family 2 subfamily J member 2 |
| Serotonergic | *CYP4X1* | 260293 | 1 | p33 | cytochrome P450 family 4 subfamily X member 1 |
| Serotonergic | *ALOX5* | 240 | 10 | q11.21 | arachidonate 5-lipoxygenase |
| Serotonergic | *ALOX12* | 239 | 17 | p13.1 | arachidonate 12-lipoxygenase, 12S type |
| Serotonergic | *ALOX12B* | 242 | 17 | p13.1 | arachidonate 12-lipoxygenase, 12R type |
| Serotonergic | *ALOX15* | 246 | 17 | p13.2 | arachidonate 15-lipoxygenase |
| Serotonergic | *ALOX15B* | 247 | 17 | p13.1 | arachidonate 15-lipoxygenase type B |
| Serotonergic | *PTGS1* | 5742 | 9 | q33.2 | prostaglandin-endoperoxide synthase 1 |
| Serotonergic | *PTGS2* | 5743 | 1 | q31.1 | prostaglandin-endoperoxide synthase 2 |
| Serotonergic | *GNAS* | 2778 | 20 | q13.32 | GNAS complex locus |
| Serotonergic | *ADCY5* | 111 | 3 | q21.1 | adenylate cyclase 5 |
| Serotonergic | *PRKACA* | 5566 | 19 | p13.12 | protein kinase cAMP-activated catalytic subunit alpha |
| Serotonergic | *PRKACB* | 5567 | 1 | p31.1 | protein kinase cAMP-activated catalytic subunit beta |
| Serotonergic | *PRKACG* | 5568 | 9 | q21.11 | protein kinase cAMP-activated catalytic subunit gamma |
| Serotonergic | *KCNN2* | 3781 | 5 | q22.3 | potassium calcium-activated channel subfamily N member 2 |
| Serotonergic | *KCND2* | 3751 | 7 | q31.31 | potassium voltage-gated channel subfamily D member 2 |
| Serotonergic | *GABRB1* | 2560 | 4 | p12 | gamma-aminobutyric acid type A receptor subunit beta1 |
| Serotonergic | *GABRB3* | 2562 | 15 | q12 | gamma-aminobutyric acid type A receptor subunit beta3 |
| Serotonergic | *GABRB2* | 2561 | 5 | q34 | gamma-aminobutyric acid type A receptor subunit beta2 |
| Serotonergic | *RAPGEF3* | 10411 | 12 | q13.11 | Rap guanine nucleotide exchange factor 3 |
| Serotonergic | *APP* | 351 | 21 | q21.3 | amyloid beta precursor protein |
| Serotonergic | *CASP3* | 836 | 4 | q35.1 | caspase 3 |
| Serotonergic | *KCNJ3* | 3760 | 2 | q24.1 | potassium inwardly rectifying channel subfamily J member 3 |
| Serotonergic | *KCNJ6* | 3763 | 21 | q22.13 | potassium inwardly rectifying channel subfamily J member 6 |
| Serotonergic | *KCNJ9* | 3765 | 1 | q23.2 | potassium inwardly rectifying channel subfamily J member 9 |
| Serotonergic | *KCNJ5* | 3762 | 11 | q24.3 | potassium inwardly rectifying channel subfamily J member 5 |
| Serotonergic | *TRPC1* | 7220 | 3 | q23 | transient receptor potential cation channel subfamily C member 1 |
| Serotonergic | *SLC6A4* | 6532 | 17 | q11.2 | solute carrier family 6 member 4 |
| Serotonergic | *MAOB* | 4129 | X | p11.3 | monoamine oxidase B |
| Serotonergic | *MAOA* | 4128 | X | p11.3 | monoamine oxidase A |
| Cholinergic | *AKT1* | 207 | 14 | q32.33 | v-akt murine thymoma viral oncogene homolog 1 |
| Cholinergic | *AKT2* | 208 | 19 | q13.2 | v-akt murine thymoma viral oncogene homolog 2 |
| Cholinergic | *AKT3* | 10000 | 1 | q44 | v-akt murine thymoma viral oncogene homolog 3 |
| Cholinergic | *ARG1* | 383 | 6 | q23.2 | arginase, live |
| Cholinergic | *ARG2* | 384 | 14 | q24.1 | arginase 2 |
| Cholinergic | *CAMK2B* | 816 | 7 | p13 | calcium/calmodulin-dependent protein kinase II beta |
| Cholinergic | *CAMK2G* | 818 | 10 | q22.2 | calcium/calmodulin-dependent protein kinase II gamma |
| Cholinergic | *CHRM1* | 1128 | 11 | q12.3 | cholinergic receptor, muscarinic 1 |
| Cholinergic | *CHRM2* | 1129 | 7 | q33 | cholinergic receptor, muscarinic 2 |
| Cholinergic | *CHRM3* | 1131 | 1 | q43 | cholinergic receptor, muscarinic 3 |
| Cholinergic | *CHRM4* | 1132 | 11 | p11.2 | cholinergic receptor, muscarinic 4 |
| Cholinergic | *CHRM5* | 1133 | 15 | q14 | cholinergic receptor, muscarinic 5 |
| Cholinergic | *CREB1* | 1385 | 2 | q33.3 | cAMP responsive element binding protein 1 |
| Cholinergic | *CREB3* | 10488 | 9 | p13.3 | cAMP responsive element binding protein 3 |
| Cholinergic | *EGFR* | 1956 | 7 | p11.2 | epidermal growth factor receptor |
| Cholinergic | *ELK1* | 2002 | X | p11.23 | ELK1, member of ETS oncogene family |
| Cholinergic | *FOS* | 2353 | 14 | q24.3 | FBJ murine osteosarcoma viral oncogene homolog |
| Cholinergic | *FYN* | 2534 | 6 | q21 | FYN oncogene related to SRC, FGR, YES |
| Cholinergic | *GNA11* | 2767 | 19 | p13.3 | guanine nucleotide binding protein (G protein), alpha 11 (Gq class) |
| Cholinergic | *GNAI2* | 2771 | 3 | p21.31 | guanine nucleotide binding protein (G protein), alpha inhibiting activity polypeptide 2 |
| Cholinergic | *GNAI3* | 2773 | 1 | p13.3 | guanine nucleotide binding protein (G protein), alpha inhibiting activity polypeptide 3 |
| Cholinergic | *GNAO1* | 2775 | 16 | q13 | guanine nucleotide binding protein (G protein), alpha activating activity polypeptide O |
| Cholinergic | *GNB1* | 2782 | 1 | p36.33 | guanine nucleotide binding protein (G protein), beta polypeptide 1 |
| Cholinergic | *GNB2* | 2783 | 7 | q22.1 | guanine nucleotide binding protein (G protein), beta polypeptide 2 |
| Cholinergic | *GNB3* | 2784 | 12 | p13.31 | guanine nucleotide binding protein (G protein), beta polypeptide 3 |
| Cholinergic | *GNB4* | 59345 | 3 | q26.33 | guanine nucleotide binding protein (G protein), beta polypeptide 4 |
| Cholinergic | *GNB5* | 10681 | 15 | q21.2 | guanine nucleotide binding protein (G protein), beta 5 |
| Cholinergic | *GNG10* | 2790 | 9 | q31.3 | guanine nucleotide binding protein (G protein), gamma 10 |
| Cholinergic | *GNG11* | 2791 | 7 | q21.3 | guanine nucleotide binding protein (G protein), gamma 11 |
| Cholinergic | *GNG12* | 55970 | 1 | p31.3 | guanine nucleotide binding protein (G protein), gamma 12 |
| Cholinergic | *GNG13* | 51764 | 16 | p13.3 | guanine nucleotide binding protein (G protein), gamma 13 |
| Cholinergic | *GNG2* | 54331 | 14 | q22.1 | guanine nucleotide binding protein (G protein), gamma 2 |
| Cholinergic | *GNG3* | 2785 | 11 | q12.3 | guanine nucleotide binding protein (G protein), gamma 3 |
| Cholinergic | *GNG4* | 2786 | 1 | q42.3 | guanine nucleotide binding protein (G protein), gamma 4 |
| Cholinergic | *GNG5* | 2787 | 1 | p22.3 | guanine nucleotide binding protein (G protein), gamma 5 |
| Cholinergic | *GNG7* | 2788 | 19 | p13.3 | guanine nucleotide binding protein (G protein), gamma 7 |
| Cholinergic | *GNG8* | 94235 | 19 | q13.32 | guanine nucleotide binding protein (G protein), gamma 8 |
| Cholinergic | *GNGT1* | 2792 | 7 | q21.3 | guanine nucleotide binding protein (G protein), gamma transducing activity polypeptide 1 |
| Cholinergic | *GNGT2* | 2793 | 17 | q21.32 | guanine nucleotide binding protein (G protein), gamma transducing activity polypeptide 2 |
| Cholinergic | *GRB2* | 2885 | 17 | q25.1 | growth factor receptor-bound protein 2 |
| Cholinergic | *HRAS* | 3265 | 11 | p15.5 | Harvey rat sarcoma viral oncogene homolog |
| Cholinergic | *ITPR1* | 3708 | 3 | p26.1 | inositol 1,4,5-trisphosphate receptor, type 1 |
| Cholinergic | *KDR* | 3791 | 4 | q12 | kinase insert domain receptor (a type III receptor tyrosine kinase) |
| Cholinergic | *KRAS* | 3845 | 12 | p12.1 | Kirsten rat sarcoma viral oncogene homolog |
| Cholinergic | *GNG14* | 1.05E+08 | 19 | p13.13 | guanine nucleotide binding protein (G protein), gamma 12-like |
| Cholinergic | *MAP2K1* | 5604 | 15 | q22.31 | mitogen-activated protein kinase kinase 1 |
| Cholinergic | *MAP2K2* | 5605 | 19 | p13.3 | mitogen-activated protein kinase kinase 2 |
| Cholinergic | *MAP3K1* | 4214 | 5 | q11.2 | mitogen-activated protein kinase kinase kinase 1, E3 ubiquitin protein ligase |
| Cholinergic | *MAP3K2* | 10746 | 2 | q14.3 | mitogen-activated protein kinase kinase kinase 2 |
| Cholinergic | *MAP3K3* | 4215 | 17 | q23.3 | mitogen-activated protein kinase kinase kinase 3 |
| Cholinergic | *MAPK1* | 5594 | 22 | q11.22 | mitogen-activated protein kinase 1 |
| Cholinergic | *MAPK3* | 5595 | 16 | p11.2 | mitogen-activated protein kinase 3 |
| Cholinergic | *MMP7* | 4316 | 11 | q22.2 | matrix metallopeptidase 7 (matrilysin, uterine) |
| Cholinergic | *MTOR* | 2475 | 1 | p36.22 | mechanistic target of rapamycin (serine/threonine kinase) |
| Cholinergic | *NFKB1* | 4790 | 4 | q24 | nuclear factor of kappa light polypeptide gene enhancer in B-cells 1 |
| Cholinergic | *NFKB2* | 4791 | 10 | q24.32 | nuclear factor of kappa light polypeptide gene enhancer in B-cells 2 (p49/p100) |
| Cholinergic | *NOS1* | 4842 | 12 | q24.22 | nitric oxide synthase 1 (neuronal) |
| Cholinergic | *NOS2* | 4843 | 17 | q11.2 | nitric oxide synthase 2, inducible |
| Cholinergic | *NOS3* | 4846 | 7 | q36.1 | nitric oxide synthase 3 (endothelial cell) |
| Cholinergic | *NRAS* | 4893 | 1 | p13.2 | neuroblastoma RAS viral (v-ras) oncogene homolog |
| Cholinergic | *PIK3C2A* | 5286 | 11 | p15.1 | phosphatidylinositol-4-phosphate 3-kinase, catalytic subunit type 2 alpha |
| Cholinergic | *PIK3C2B* | 5287 | 1 | q32.1 | phosphatidylinositol-4-phosphate 3-kinase, catalytic subunit type 2 beta |
| Cholinergic | *PIK3C2G* | 5288 | 12 | p12.3 | phosphatidylinositol-4-phosphate 3-kinase, catalytic subunit type 2 gamma |
| Cholinergic | *PIK3C3* | 5289 | 18 | q12.3 | phosphatidylinositol 3-kinase, catalytic subunit type 3 |
| Cholinergic | *PIK3CA* | 5290 | 3 | q26.32 | phosphatidylinositol-4,5-bisphosphate 3-kinase, catalytic subunit alpha |
| Cholinergic | *PIK3CB* | 5291 | 3 | q22.3 | phosphatidylinositol-4,5-bisphosphate 3-kinase, catalytic subunit beta |
| Cholinergic | *PIK3CD* | 5293 | 1 | p36.22 | phosphatidylinositol-4,5-bisphosphate 3-kinase, catalytic subunit delta |
| Cholinergic | *PIK3CG* | 5294 | 7 | q22.3 | phosphatidylinositol-4,5-bisphosphate 3-kinase, catalytic subunit gamma |
| Cholinergic | *PIK3R1* | 5295 | 5 | q13.1 | phosphoinositide-3-kinase, regulatory subunit 1 (alpha) |
| Cholinergic | *PIK3R2* | 5296 | 19 | p13.11 | phosphoinositide-3-kinase, regulatory subunit 2 (beta) |
| Cholinergic | *PIK3R3* | 8503 | 1 | p34.1 | phosphoinositide-3-kinase, regulatory subunit 3 (gamma) |
| Cholinergic | *PIK3R5* | 23533 | 17 | p13.1 | phosphoinositide-3-kinase, regulatory subunit 5 |
| Cholinergic | *PIK3R6* | 146850 | 17 | p13.1 | phosphoinositide-3-kinase, regulatory subunit 6 |
| Cholinergic | *PLA2G10* | 8399 | 16 | p13.12 | phospholipase A2, group X |
| Cholinergic | *PLA2G12A* | 81579 | 4 | q25 | phospholipase A2, group XIIA |
| Cholinergic | *PLA2G12B* | 84647 | 10 | q22.1 | phospholipase A2, group XIIB |
| Cholinergic | *PLAAT3* | 11145 | 11 | q13.1 | phospholipase A2, group XVI |
| Cholinergic | *PLA2G2C* | 391013 | 1 | p36.12 | phospholipase A2, group IIC |
| Cholinergic | *PLA2G2D* | 26279 | 1 | p36.12 | phospholipase A2, group IID |
| Cholinergic | *PLA2G2E* | 30814 | 1 | p36.13 | phospholipase A2, group IIE |
| Cholinergic | *PLA2G2F* | 64600 | 1 | p36.12 | phospholipase A2, group IIF |
| Cholinergic | *PLA2G3* | 50487 | 22 | q12.2 | phospholipase A2, group III |
| Cholinergic | *PLA2G4A* | 5321 | 1 | q31.1 | phospholipase A2, group IVA (cytosolic, calcium-dependent) |
| Cholinergic | *PLA2G4C* | 8605 | 19 | q13.33 | phospholipase A2, group IVC (cytosolic, calcium-independent) |
| Cholinergic | *PLA2G4D* | 283748 | 15 | q15.1 | phospholipase A2, group IVD (cytosolic) |
| Cholinergic | *PLA2G5* | 5322 | 1 | p36.13 | phospholipase A2, group V |
| Cholinergic | *PLA2G6* | 8398 | 22 | q13.1 | phospholipase A2, group VI (cytosolic, calcium-independent) |
| Cholinergic | *PLCB1* | 23236 | 20 | p12.3 | phospholipase C, beta 1 (phosphoinositide-specific) |
| Cholinergic | *PLCB2* | 5330 | 15 | q15.1 | phospholipase C, beta 2 |
| Cholinergic | *PLCB3* | 5331 | 11 | q13.1 | phospholipase C, beta 3 (phosphatidylinositol-specific) |
| Cholinergic | *PLCB4* | 5332 | 20 | p12.3 | phospholipase C, beta 4 |
| Cholinergic | *PRKCA* | 5578 | 17 | q24.2 | protein kinase C, alpha |
| Cholinergic | *PRKCD* | 5580 | 3 | p21.1 | protein kinase C, delta |
| Cholinergic | *PRKCE* | 5581 | 2 | p21 | protein kinase C, epsilon |
| Cholinergic | *PTGER2* | 5732 | 14 | q22.1 | prostaglandin E receptor 2 (subtype EP2), 53kDa |
| Cholinergic | *PTGS1* | 5742 | 9 | q33.2 | prostaglandin-endoperoxide synthase 1 (prostaglandin G/H synthase and cyclooxygenase) |
| Cholinergic | *PTGS2* | 5743 | 1 | q31.1 | prostaglandin-endoperoxide synthase 2 (prostaglandin G/H synthase and cyclooxygenase) |
| Cholinergic | *PTK2B* | 2185 | 8 | p21.2 | PTK2B protein tyrosine kinase 2 beta |
| Cholinergic | *RAF1* | 5894 | 3 | p25.2 | v-raf-1 murine leukemia viral oncogene homolog 1 |
| Cholinergic | *RAP1A* | 5906 | 1 | p13.2 | RAP1A, member of RAS oncogene family |
| Cholinergic | *RAP1GAP* | 5909 | 1 | p36.12 | RAP1 GTPase activating protein |
| Cholinergic | *RPS6KA1* | 6195 | 1 | p36.11 | ribosomal protein S6 kinase, 90kDa, polypeptide 1 |
| Cholinergic | *RPS6KB1* | 6198 | 17 | q23.1 | ribosomal protein S6 kinase, 70kDa, polypeptide 1 |
| Cholinergic | *RPS6KB2* | 6199 | 11 | q13.2 | ribosomal protein S6 kinase, 70kDa, polypeptide 2 |
| Cholinergic | *SHC1* | 6464 | 1 | q21.3 | SHC (Src homology 2 domain containing) transforming protein 1 |
| Cholinergic | *SHC2* | 25759 | 19 | p13.3 | SHC (Src homology 2 domain containing) transforming protein 2 |
| Cholinergic | *SHC3* | 53358 | 9 | q22.1 | SHC (Src homology 2 domain containing) transforming protein 3 |
| Cholinergic | *SHC4* | 399694 | 15 | q21.1 | SHC (Src homology 2 domain containing) family, member 4 |
| Cholinergic | *SOS1* | 6654 | 2 | p22.1 | son of sevenless homolog 1 (Drosophila) |
| Cholinergic | *SOS2* | 6655 | 14 | q21.3 | son of sevenless homolog 2 (Drosophila) |
| Cholinergic | *SRC* | 6714 | 20 | q11.23 | v-src sarcoma (Schmidt-Ruppin A-2) viral oncogene homolog (avian) |
| Cholinergic | *VEGFA* | 7422 | 6 | p21.1 | vascular endothelial growth factor A |
| Cholinergic | *CHAT* | 1103 | 10 | q11.23 | choline O-acetyltransferase |
| Cholinergic | *ACHE* | 43 | 7 | q22.1 | acetylcholinesterase (Cartwright blood group) |
| Cholinergic | *SLC18A3* | 6572 | 10 | q11.23 | solute carrier family 18 member A3 |
| Cholinergic | *GNAQ* | 2776 | 9 | q21.2 | G protein subunit alpha q |
| Cholinergic | *ITPR2* | 3709 | 12 | p11.23 | inositol 1,4,5-trisphosphate receptor type 2 |
| Cholinergic | *ITPR3* | 3710 | 6 | p21.31 | inositol 1,4,5-trisphosphate receptor type 3 |
| Cholinergic | *PRKCB* | 5579 | 16 | p12.2 | protein kinase C beta |
| Cholinergic | *PRKCG* | 5582 | 19 | q13.42 | protein kinase C gamma |
| Cholinergic | *KCNQ1* | 3784 | 11 | p15.5 | potassium voltage-gated channel subfamily Q member 1 |
| Cholinergic | *KCNQ2* | 3785 | 20 | q13.33 | potassium voltage-gated channel subfamily Q member 2 |
| Cholinergic | *KCNQ3* | 3786 | 8 | q24.22 | potassium voltage-gated channel subfamily Q member 3 |
| Cholinergic | *KCNQ4* | 9132 | 1 | p34.2 | potassium voltage-gated channel subfamily Q member 4 |
| Cholinergic | *KCNQ5* | 56479 | 6 | q13 | potassium voltage-gated channel subfamily Q member 5 |
| Cholinergic | *KCNJ2* | 3759 | 17 | q24.3 | potassium inwardly rectifying channel subfamily J member 2 |
| Cholinergic | *KCNJ12* | 3768 | 17 | p11.2 | potassium inwardly rectifying channel subfamily J member 12 |
| Cholinergic | *KCNJ18* | 1E+08 | 17 | p11.2 | potassium inwardly rectifying channel subfamily J member 18 |
| Cholinergic | *KCNJ4* | 3761 | 22 | q13.1 | potassium inwardly rectifying channel subfamily J member 4 |
| Cholinergic | *KCNJ14* | 3770 | 19 | q13.33 | potassium inwardly rectifying channel subfamily J member 14 |
| Cholinergic | *GNAI1* | 2770 | 7 | q21.11 | G protein subunit alpha i1 |
| Cholinergic | *KCNJ3* | 3760 | 2 | q24.1 | potassium inwardly rectifying channel subfamily J member 3 |
| Cholinergic | *KCNJ6* | 3763 | 21 | q22.13 | potassium inwardly rectifying channel subfamily J member 6 |
| Cholinergic | *CHRNA7* | 1139 | 15 | q13.3 | cholinergic receptor nicotinic alpha 7 subunit |
| Cholinergic | *CHRNA4* | 1137 | 20 | q13.33 | cholinergic receptor nicotinic alpha 4 subunit |
| Cholinergic | *CHRNB2* | 1141 | 1 | q21.3 | cholinergic receptor nicotinic beta 2 subunit |
| Cholinergic | *CHRNA3* | 1136 | 15 | q25.1 | cholinergic receptor nicotinic alpha 3 subunit |
| Cholinergic | *CHRNB4* | 1143 | 15 | q25.1 | cholinergic receptor nicotinic beta 4 subunit |
| Cholinergic | *CHRNA6* | 8973 | 8 | p11.21 | cholinergic receptor nicotinic alpha 6 subunit |
| Cholinergic | *ADCY1* | 107 | 7 | p12.3 | adenylate cyclase 1 |
| Cholinergic | *ADCY2* | 108 | 5 | p15.31 | adenylate cyclase 2 |
| Cholinergic | *ADCY3* | 109 | 2 | p23.3 | adenylate cyclase 3 |
| Cholinergic | *ADCY4* | 196883 | 14 | q12 | adenylate cyclase 4 |
| Cholinergic | *ADCY5* | 111 | 3 | q21.1 | adenylate cyclase 5 |
| Cholinergic | *ADCY6* | 112 | 12 | q13.12 | adenylate cyclase 6 |
| Cholinergic | *ADCY7* | 113 | 16 | q12.1 | adenylate cyclase 7 |
| Cholinergic | *ADCY8* | 114 | 8 | q24.22 | adenylate cyclase 8 |
| Cholinergic | *ADCY9* | 115 | 16 | p13.3 | adenylate cyclase 9 |
| Cholinergic | *PRKACA* | 5566 | 19 | p13.12 | protein kinase cAMP-activated catalytic subunit alpha |
| Cholinergic | *PRKACB* | 5567 | 1 | p31.1 | protein kinase cAMP-activated catalytic subunit beta |
| Cholinergic | *PRKACG* | 5568 | 9 | q21.11 | protein kinase cAMP-activated catalytic subunit gamma |
| Cholinergic | *ATF4* | 468 | 22 | q13.1 | activating transcription factor 4 |
| Cholinergic | *CREB3L1* | 90993 | 11 | p11.2 | cAMP responsive element binding protein 3 like 1 |
| Cholinergic | *CREB3L2* | 64764 | 7 | q33 | cAMP responsive element binding protein 3 like 2 |
| Cholinergic | *CREB3L3* | 84699 | 19 | p13.3 | cAMP responsive element binding protein 3 like 3 |
| Cholinergic | *CREB3L4* | 148327 | 1 | q21.3 | cAMP responsive element binding protein 3 like 4 |
| Cholinergic | *CREB5* | 9586 | 7 | p15.1 | cAMP responsive element binding protein 5 |
| Cholinergic | *CAMK2A* | 815 | 5 | q32 | calcium/calmodulin dependent protein kinase II alpha |
| Cholinergic | *CAMK2D* | 817 | 4 | q26 | calcium/calmodulin dependent protein kinase II delta |
| Cholinergic | *CAMK4* | 814 | 5 | q22.1 | calcium/calmodulin dependent protein kinase IV |
| Cholinergic | *JAK2* | 3717 | 9 | p24.1 | Janus kinase 2 |
| Cholinergic | *BCL2* | 596 | 18 | q21.33 | BCL2 apoptosis regulator |
| Cholinergic | *CACNA1A* | 773 | 19 | p13.13 | calcium voltage-gated channel subunit alpha1 A |
| Cholinergic | *CACNA1B* | 774 | 9 | q34.3 | calcium voltage-gated channel subunit alpha1 B |
| Cholinergic | *CACNA1C* | 775 | 12 | p13.33 | calcium voltage-gated channel subunit alpha1 C |
| Cholinergic | *CACNA1D* | 776 | 3 | p21.1 | calcium voltage-gated channel subunit alpha1 D |
| Cholinergic | *CACNA1F* | 778 | X | p11.23 | calcium voltage-gated channel subunit alpha1 F |
| Cholinergic | *CACNA1S* | 779 | 1 | q32.1 | calcium voltage-gated channel subunit alpha1 S |
| Cholinergic | *SLC5A7* | 60482 | 2 | q12.3 | solute carrier family 5 member 7 |
| Cholinergic | *EGF* | 1950 | 4 | q25 | epidermal growth factor |
| Cholinergic | *PDGFA* | 5154 | 7 | p22.3 | platelet derived growth factor subunit A |
| Cholinergic | *PDGFB* | 5155 | 22 | q13.1 | platelet derived growth factor subunit B |
| Cholinergic | *PDGFC* | 56034 | 4 | q32.1 | platelet derived growth factor C |
| Cholinergic | *PDGFD* | 80310 | 11 | q22.3 | platelet derived growth factor D |
| Cholinergic | *PDGFRA* | 5156 | 4 | q12 | platelet derived growth factor receptor alpha |
| Cholinergic | *PDGFRB* | 5159 | 5 | q32 | platelet derived growth factor receptor beta |
| Cholinergic | *RALGDS* | 5900 | 9 | q34.2 | ral guanine nucleotide dissociation stimulator |
| Cholinergic | *MAPK8* | 5599 | 10 | q11.22 | mitogen-activated protein kinase 8 |
| Cholinergic | *MAPK10* | 5602 | 4 | q21.3 | mitogen-activated protein kinase 10 |
| Cholinergic | *MAPK9* | 5601 | 5 | q35.3 | mitogen-activated protein kinase 9 |
| Cholinergic | *PLA2G4E* | 123745 | 15 | q15.1 | phospholipase A2 group IVE |
| Cholinergic | *JMJD7_PLA2G4B* | 8681 | 15 | q15.1 | JMJD7-PLA2G4B readthrough |
| Cholinergic | *PLA2G4B* | 1E+08 | 15 | q15.1 | phospholipase A2 group IVB |
| Cholinergic | *PLA2G4F* | 255189 | 15 | q15.1 | phospholipase A2 group IVF |
| Cholinergic | *PDPK1* | 5170 | 16 | p13.3 | 3-phosphoinositide dependent protein kinase 1 |
| Cholinergic | *TSC1* | 7248 | 9 | q34.13 | TSC complex subunit 1 |
| Cholinergic | *TSC2* | 7249 | 16 | p13.3 | TSC complex subunit 2 |
| Cholinergic | *RHEB* | 6009 | 7 | q36.1 | Ras homolog, mTORC1 binding |
| Cholinergic | *EIF4EBP1* | 1978 | 8 | p11.23 | eukaryotic translation initiation factor 4E binding protein 1 |
| Cholinergic | *PIP5K1C* | 23396 | 19 | p13.3 | phosphatidylinositol-4-phosphate 5-kinase type 1 gamma |
| Cholinergic | *PIP5K1A* | 8394 | 1 | q21.3 | phosphatidylinositol-4-phosphate 5-kinase type 1 alpha |
| Cholinergic | *PIP5K1B* | 8395 | 9 | q21.11 | phosphatidylinositol-4-phosphate 5-kinase type 1 beta |
| Cholinergic | *WAS* | 7454 | X | p11.23 | WASP actin nucleation promoting factor |
| Cholinergic | *RAC1* | 5879 | 7 | p22.1 | Rac family small GTPase 1 |
| Cholinergic | *RAC2* | 5880 | 22 | q13.1 | Rac family small GTPase 2 |
| Cholinergic | *RAC3* | 5881 | 17 | q25.3 | Rac family small GTPase 3 |
| Cholinergic | *WASF1* | 8936 | 6 | q21 | WASP family member 1 |
| Cholinergic | *WASF2* | 10163 | 1 | p36.11 | WASP family member 2 |
| Cholinergic | *WASF3* | 10810 | 13 | q12.13 | WASP family member 3 |
| Cholinergic | *SP1* | 6667 | 12 | q13.13 | Sp1 transcription factor |
| Cholinergic | *PLD1* | 5337 | 3 | q26.31 | phospholipase D1 |
| Cholinergic | *PLD2* | 5338 | 17 | p13.2 | phospholipase D2 |
| Cholinergic | *SLC44A1* | 23446 | 9 | q31.1 | solute carrier family 44 member 1 |
| Cholinergic | *SLC44A4* | 80736 | 6 | p21.33 | solute carrier family 44 member 4 |
| Cholinergic | *SLC44A5* | 204962 | 1 | p31.1 | solute carrier family 44 member 5 |
| Cholinergic | *SLC44A2* | 57153 | 19 | p13.2 | solute carrier family 44 member 2 |
| Cholinergic | *SLC44A3* | 126969 | 1 | p21.3 | solute carrier family 44 member 3 |
| Cholinergic | *SLC22A1* | 6580 | 6 | q25.3 | solute carrier family 22 member 1 |
| Cholinergic | *SLC22A2* | 6582 | 6 | q25.3 | solute carrier family 22 member 2 |
| Cholinergic | *SLC22A3* | 6581 | 6 | q25.3 | solute carrier family 22 member 3 |
| Cholinergic | *SLC22A5* | 6584 | 5 | q31.1 | solute carrier family 22 member 5 |
| Cholinergic | *SLC22A4* | 6583 | 5 | q31.1 | solute carrier family 22 member 4 |
| Cholinergic | *CHKA* | 1119 | 11 | q13.2 | choline kinase alpha |
| Cholinergic | *CHKB* | 1120 | 22 | q13.33 | choline kinase beta |
| Cholinergic | *HIF1A* | 3091 | 14 | q23.2 | hypoxia inducible factor 1 subunit alpha |
| Cholinergic | *JUN* | 3725 | 1 | p32.1 | Jun proto-oncogene, AP-1 transcription factor subunit |
| Cholinergic | *PCYT1B* | 9468 | X | p22.11 | phosphate cytidylyltransferase 1B, choline |
| Cholinergic | *PCYT1A* | 5130 | 3 | q29 | phosphate cytidylyltransferase 1A, choline |
| Cholinergic | *CHPT1* | 56994 | 12 | q23.2 | choline phosphotransferase 1 |
| Cholinergic | *PLCG1* | 5335 | 20 | q12 | phospholipase C gamma 1 |
| Cholinergic | *PLPP1* | 8611 | 5 | q11.2 | phospholipid phosphatase 1 |
| Cholinergic | *PLPP3* | 8613 | 1 | p32.2 | phospholipid phosphatase 3 |
| Cholinergic | *PLPP2* | 8612 | 19 | p13.3 | phospholipid phosphatase 2 |
| Cholinergic | *DGKZ* | 8525 | 11 | p11.2 | diacylglycerol kinase zeta |
| Cholinergic | *DGKD* | 8527 | 2 | q37.1 | diacylglycerol kinase delta |
| Cholinergic | *DGKI* | 9162 | 7 | q33 | diacylglycerol kinase iota |
| Cholinergic | *DGKA* | 1606 | 12 | q13.2 | diacylglycerol kinase alpha |
| Cholinergic | *DGKE* | 8526 | 17 | q22 | diacylglycerol kinase epsilon |
| Cholinergic | *DGKB* | 1607 | 7 | p21.2 | diacylglycerol kinase beta |
| Cholinergic | *DGKH* | 160851 | 13 | q14.11 | diacylglycerol kinase eta |
| Cholinergic | *DGKG* | 1608 | 3 | q27.3 | diacylglycerol kinase gamma |
| Cholinergic | *DGKQ* | 1609 | 4 | p16.3 | diacylglycerol kinase theta |
| Cholinergic | *DGKK* | 139189 | X | p11.22 | diacylglycerol kinase kappa |
| Cholinergic | *LYPLA1* | 10434 | 8 | q11.23 | lysophospholipase 1 |
| Cholinergic | *GPCPD1* | 56261 | 20 | p12.3 | glycerophosphocholine phosphodiesterase 1 |

# Table S2. Clinical characteristics of breast cancer patients from TCGA and WCH cohort, separately

|  | West China Hospital (WCH) cohort | | | The Cancer Genome Atlas (TCGA) | | |
| --- | --- | --- | --- | --- | --- | --- |
|  | Cases *^a^* (N=96) | Controls (N=96) | Whole cohort  (N = 7784) | Cases (N=112) | Controls (N=112) | Whole cohort (N = 1063) |
| Age at diagnosis |  |  |  |  |  |  |
| Mean (SD) | 50 (12) | 50 (10) | 48 (10) | 60 (15) | 54 (11) | 58 (13) |
| Range | 23 - 84 | 27 - 74 | 18 - 94 | 26 - 90 | 27 - 83 | 26 - 90 |
|  | N (%) | N (%) | N (%) | N (%) | N (%) | N (%) |
| Race/Ethnicity |  |  |  |  |  |  |
| Asian | - | - | - | 4 (3.6) | 4 (3.6) | 61 (5.7) |
| Han | 93 (96.9) | 96 (100) | 7607(97.7) | - | - | - |
| Other | 3 (3.1) | 0 (0) | 177 (2.3) | - | - | - |
| Black or African American | - | - | - | 17 (15.2) | 18 (16.1) | 175 (16.5) |
| Other | - | - | - | 8 (7.1) | 3 (2.7) | 91 (8.6) |
|  |  |  |  |  |  |  |
| White | - | - | - | 83 (74.1) | 87 (77.7) | 736 (69.2) |
| Menopausal status at diagnosis | | | |  |  |  |
| Premenopausal | 47 (49.0) | 60 (62.5) | 4716 (60.6) | 22 (19.6) | 41 (36.6) | 225 (21.2) |
| Postmenopausal | 49 (51.0) | 36 (37.5) | 3052 (39.2) | 72 (64.3) | 62 (55.4) | 694 (65.3) |
| Unknown | 0 (0.0) | 0 (0.0) | 16 (0.2) | 18 (16.1) | 9 (8.0) | 144 (13.5) |
| Stage |  |  |  |  |  |  |
| Stage I | 9 (9.4) | 17 (17.7) | 1695 (21.8) | 10 (8.9) | 28 (25.0) | 188 (17.7) |
| Stage II | 42 (43.8) | 60 (62.5) | 3599 (46.2) | 58 (51.8) | 67 (59.8) | 611 (57.5) |
| Stage III | 45 (46.9) | 19 (19.8) | 2028 (26.1) | 44 (39.3) | 17 (15.2) | 248 (23.3) |
| Unknown | 0 (0.0) | 0 (0.0) | 462 (5.9) | 0 (0.0) | 0 (0.0) | 16 (1.5) |
| Molecular subtype |  |  |  |  |  |  |
| Luminal A | 2 (2.1) | 3 (3.1) | 950 (12.2) | 36 (32.1) | 44 (39.3) | 439 (41.3) |
| Luminal B | 52 (54.2) | 53 (55.2) | 4292 (55.1) | 31 (27.7) | 33 (29.5) | 274 (25.8) |
| TNBC | 14 (14.6) | 15 (15.6) | 1045 (13.4) | 23 (20.5) | 27 (24.1) | 156 (14.7) |
| HER2-enriched | 18 (18.8) | 19 (19.8) | 840 (10.8) | 5 (4.5) | 7 (6.2) | 40 (3.8) |
| Unclassified | 10 (10.4) | 6 (6.2) | 657 (8.4) | 17 (15.2) | 1 (0.9) | 154 (14.5) |
| Estrogen receptor, ER |  |  |  |  |  |  |
| Negative | 41 (42.7) | 40 (41.7) | 2414 (31.0) | 34 (30.4) | 35 (31.2) | 234 (22.0) |
| Positive | 55 (57.3) | 56 (58.3) | 5243 (67.4) | 78 (69.6) | 77 (68.8) | 780 (73.4) |
| Unknown | 0 (0.0) | 0 (0.0) | 127 (1.6) | 0 (0.0) | 0 (0.0) | 49 (4.6) |
| Progesterone receptor, PR |  |  |  |  |  |  |
| Negative | 45 (46.9) | 42 (43.8) | 2813 (36.1) | 51 (45.5) | 49 (43.8) | 333 (31.3) |
| Positive | 51 (53.1) | 54 (56.2) | 4844 (62.2) | 61 (54.5) | 63 (56.2) | 677 (63.7) |
| Unknown | 0 (0.0) | 0 (0.0) | 127 (1.6) | 0 (0.0) | 0 (0.0) | 53 (5.0) |
| Human epidermal growth factor receptor-2, HER2 | | | |  |  |  |
| Negative | 49 (51.0) | 54 (56.2) | 4760 (61.2) | 74 (66.1) | 89 (79.5) | 742 (69.8) |
| Positive | 39 (40.6) | 42 (43.8) | 1889 (24.3) | 22 (19.6) | 23 (20.5) | 188 (17.7) |
| Unknown | 8 (8.3) | 0 (0.0) | 1135 (14.6) | 16 (14.3) | 0 (0.0) | 133 (12.5) |
| Primary surgery |  |  |  |  |  |  |
| Breast conserving | 1 (1.0) | 4 (4.2) | 521 (6.7) | 22 (19.6) | 31 (27.7) | 243 (22.9) |
| Mastectomy | 94 (97.9) | 92 (95.8) | 7025 (90.2) | 54 (48.2) | 46 (41.1) | 500 (47.0) |
| Unknown | 1 (1.0) | 0 (0.0) | 238 (3.1) | 36 (32.1) | 35 (31.2) | 320 (30.1) |
| Chemotherapy *^b^* |  |  |  |  |  |  |
| No | 6 (6.2) | 1 (1.0) | 515 (6.6) | 17 (15.2) | 8 (7.1) | 191 (18.0) |
| Yes | 90 (93.8) | 95 (99.0) | 7269 (93.4) | 49 (43.8) | 91 (81.2) | 566 (53.2) |
| Unknown | 0 (0.0) | 0 (0.0) | 0 (0.0) | 46 (41.1) | 13 (11.6) | 306 (28.8) |
| Radiotherapy |  |  |  |  |  |  |
| No | 57 (59.4) | 66 (68.8) | 5362 (68.9) | 46 (41.1) | 31 (27.7) | 424 (39.9) |
| Yes | 39 (40.6) | 30 (31.2) | 2422 (31.1) | 47 (42.0) | 81 (72.3) | 547 (51.5) |
| Unknown | 0 (0.0) | 0 (0.0) | 0 (0.0) | 19 (17.0) | 0 (0.0) | 92 (8.7) |
| Hormonal therapy *^b^* |  |  |  |  |  |  |
| No | 43 (44.8) | 34 (35.4) | 2658 (34.1) | 21 (18.8) | 46 (41.1) | 249 (23.4) |
| Yes | 53 (55.2) | 62 (64.6) | 5126 (65.9) | 45 (40.2) | 53 (47.3) | 508 (47.8) |
| Unknown | 0 (0.0) | 0 (0.0) | 0 (0.0) | 46 (41.1) | 13 (11.6) | 306 (28.8) |

^a^ Cases were patients with any invasive disease-free survival (iDFS) endpoints during the first five years after cancer diagnosis, and controls were patients who survived at least first five years and had no iDFS endpoints through the last follow-up.

*^b^* Patients with at least one record of drugs (chemotherapy, hormonal therapy or targeted molecular therapy) but lacking records of the specific therapy were classified as “No”. Information on chemotherapy, radiotherapy, or hormonal therapy in TCGA cohort is not complete.

# Table S3. The associations between tumor mutation burden (TMB) of neuroendocrine pathways and prognosis, stratification analysis by ER status

| Pathway | Somatic Mutation ^a^ | ER positive (N=266) | | | ER negative (N=150) | | |
| --- | --- | --- | --- | --- | --- | --- | --- |
|  |  | Cases | Controls | Odds ratio ^b^ | Cases | Controls | Odds ratio |
| Adrenergic | No mutation | 42 | 49 | Ref. | 35 | 26 | Ref. |
|  | Any Mutation | 91 | 84 | 1.53 (0.87, 2.69) | 40 | 49 | 0.59 (0.28, 1.27) |
|  | TMB | 133 | 133 | 1.09 (0.89, 1.32) | 75 | 75 | 0.97 (0.74, 1.26) |
| Glucocorticoid | No mutation | 80 | 88 | Ref. | 42 | 49 | Ref. |
|  | Any Mutation | 53 | 45 | 1.55 (0.9, 2.67) | 33 | 26 | 2.41 (1.07, 5.41) |
|  | TMB | 133 | 133 | 1.13 (0.94, 1.34) | 75 | 75 | 1.34 (1.04, 1.73) |
| Dopaminergic | No mutation | 89 | 88 | Ref. | 46 | 50 | Ref. |
|  | Any Mutation | 44 | 45 | 0.82 (0.47, 1.42) | 29 | 25 | 1.48 (0.68, 3.22) |
|  | TMB | 133 | 133 | 0.94 (0.79, 1.11) | 75 | 75 | 1.17 (0.91, 1.5) |
| Serotonergic | No mutation | 89 | 93 | Ref. | 46 | 47 | Ref. |
|  | Any Mutation | 44 | 40 | 1.02 (0.59, 1.79) | 29 | 28 | 1.42 (0.64, 3.13) |
|  | TMB | 133 | 133 | 1.02 (0.85, 1.22) | 75 | 75 | 1.1 (0.86, 1.41) |
| Cholinergic | No mutation | 38 | 50 | Ref. | 23 | 26 | Ref. |
|  | Any Mutation | 95 | 83 | 1.69 (0.96, 2.99) | 52 | 49 | 1.53 (0.67, 3.46) |
|  | TMB | 133 | 133 | 1.13 (0.92, 1.38) | 75 | 75 | 1.16 (0.88, 1.54) |

^a^ Somatic mutation burden of candidate genes in each pathway were firstly categorized as binary variable (nonmutated group was used as reference group), and then treated as a continuous variable.

^b^ Odds ratios were estimated using logistic regression models and adjusted for cohort membership, age at diagnosis, menopausal status at diagnosis, molecular subtype, and cancer stage.

# Table S4. The associations between tumor mutation burden (TMB) of neuroendocrine pathways and prognosis with different adjustment

| Pathway | Somatic Mutation | WCH + TCGA | | WCH | | TCGA | |
| --- | --- | --- | --- | --- | --- | --- | --- |
|  |  | Basic model ^a^ | Advanced model ^b^ | Basic model ^a^ | Advanced model ^b^ | Basic model ^a^ | Advanced model ^b^ |
| Adrenergic | No mutation | Ref. | Ref. | Ref. | Ref. | Ref. | Ref. |
|  | Any Mutation | 1.04 (0.69, 1.57) | 1.21 (0.75, 1.95) | 1.73 (0.96, 3.12) | 2.04 (1.05, 3.98) | 0.58 (0.32, 1.07) | 0.75 (0.35, 1.62) |
|  | TMB | 1.04 (0.90, 1.2) | 1.08 (0.91, 1.27) | 1.21 (0.98, 1.49) | 1.26 (1, 1.59) | 0.88 (0.71, 1.09) | 0.94 (0.72, 1.22) |
| Glucocorticoid | No mutation | Ref. | Ref. | Ref. | Ref. | Ref. | Ref. |
|  | Any Mutation | 1.45 (0.97, 2.19) | 1.90 (1.18, 3.05) | 1.63 (0.89, 2.98) | 2.02 (1.03, 3.97) | 1.3 (0.74, 2.29) | 2.39 (1.14, 5.02) |
|  | TMB | 1.13 (0.99, 1.29) | 1.22 (1.05, 1.42) | 1.18 (0.97, 1.43) | 1.26 (1.01, 1.56) | 1.09 (0.91, 1.3) | 1.26 (1, 1.61) |
| Dopaminergic | No mutation | Ref. | Ref. | Ref. | Ref. | Ref. | Ref. |
|  | Any Mutation | 1.15 (0.76, 1.74) | 0.99 (0.62, 1.59) | 1.65 (0.89, 3.05) | 1.83 (0.92, 3.66) | 0.83 (0.47, 1.48) | 0.66 (0.32, 1.37) |
|  | TMB | 1.04 (0.91, 1.18) | 1 (0.87, 1.16) | 1.15 (0.95, 1.39) | 1.19 (0.96, 1.46) | 0.94 (0.79, 1.13) | 0.88 (0.7, 1.11) |
| Serotonergic | No mutation | Ref. | Ref. | Ref. | Ref. | Ref. | Ref. |
|  | Any Mutation | 1.12 (0.74, 1.69) | 1.11 (0.69, 1.79) | 1.63 (0.88, 3.03) | 1.77 (0.89, 3.52) | 0.81 (0.45, 1.44) | 0.68 (0.33, 1.43) |
|  | TMB | 1.04 (0.91, 1.18) | 1.04 (0.9, 1.21) | 1.17 (0.96, 1.41) | 1.18 (0.96, 1.46) | 0.94 (0.78, 1.13) | 0.89 (0.7, 1.13) |
| Cholinergic | No mutation | Ref. | Ref. | Ref. | Ref. | Ref. | Ref. |
|  | Any Mutation | 1.46 (0.95, 2.23) | 1.73 (1.05, 2.85) | 2.38 (1.31, 4.33) | 3.44 (1.69, 7.02) | 0.83 (0.45, 1.55) | 1.19 (0.52, 2.75) |
|  | TMB | 1.12 (0.96, 1.3) | 1.17 (0.98, 1.39) | 1.29 (1.04, 1.6) | 1.38 (1.09, 1.76) | 0.96 (0.77, 1.19) | 1.08 (0.81, 1.45) |

^a^ Odds ratios were estimated using logistic models adjusting for cohort membership and molecular subtype.

^b^ In advanced model**,** odds ratios were estimated using logistic regression and adjusted for cohort membership, molecular subtype, age at diagnosis, menopausal status at diagnosis, cancer stage, primary surgery, radiotherapy, chemotherapy, and hormonal therapy. Information on chemotherapy, radiotherapy, or hormonal therapy in TCGA cohort is incomplete

# Table S5. The associations between tumor mutation burden (TMB) of neuroendocrine pathways and prognosis, in subsets of cohorts or exclusive gene list

| Pathway | Somatic Mutation | Exclude non-breast-cancer death ^a^ | | | Exclude neoadjuvant chemotherapy ^b^ | | | Remove overlapping genes ^c^ | | |
| --- | --- | --- | --- | --- | --- | --- | --- | --- | --- | --- |
|  |  | Cases | Controls | Odds ratios | Cases | Controls | Odds ratios | Cases | Controls | Odds ratios |
| Adrenergic | No mutation | 73 | 69 | Ref. | 72 | 73 | Ref. | 147 | 144 | Ref. |
|  | Any Mutation | 123 | 127 | 1.09 (0.69, 1.72) | 124 | 123 | 1.28 (0.81, 2.03) | 61 | 64 | 1.03 (0.65, 1.65) |
|  | TMB | 196 | 196 | 1.05 (0.9, 1.23) | 196 | 196 | 1.09 (0.93, 1.28) | 208 | 208 | 1.01 (0.88, 1.16) |
| Glucocorticoid | No mutation | 114 | 129 | Ref. | 116 | 28 | Ref. | 156 | 171 | Ref. |
|  | Any Mutation | 82 | 67 | 1.69 (1.08, 2.65) | 80 | 68 | 1.58 (1.01, 2.48) | 52 | 37 | 1.87 (1.11, 3.14) |
|  | TMB | 196 | 196 | 1.17 (1.02, 1.36) | 196 | 196 | 1.16 (1, 1.34) | 208 | 208 | 1.18 (1.03, 1.35) |
| Dopaminergic | No mutation | 130 | 131 | Ref. | 127 | 131 | Ref. | 177 | 178 | Ref. |
|  | Any Mutation | 66 | 65 | 1.06 (0.67, 1.67) | 69 | 65 | 1.11 (0.71, 1.74) | 31 | 30 | 1.09 (0.6, 1.96) |
|  | TMB | 196 | 196 | 1.02 (0.89, 1.18) | 196 | 196 | 1.03 (0.9, 1.19) | 208 | 208 | 1.03 (0.9, 1.17) |
| Serotonergic | No mutation | 128 | 130 | Ref. | 127 | 132 | Ref. | 181 | 175 | Ref. |
|  | Any Mutation | 68 | 66 | 1 (0.63, 1.57) | 69 | 64 | 1.04 (0.66, 1.65) | 27 | 33 | 0.75 (0.4, 1.39) |
|  | TMB | 196 | 196 | 1.01 (0.88, 1.17) | 196 | 196 | 1.02 (0.88, 1.18) | 208 | 208 | 0.95 (0.84, 1.08) |
| Cholinergic | No mutation | 58 | 71 | Ref. | 57 | 73 | Ref. | 138 | 141 | Ref. |
|  | Any Mutation | 138 | 125 | 1.64 (1.02, 2.63) | 139 | 123 | 1.7 (1.06, 2.73) | 70 | 67 | 1.1 (0.7, 1.71) |
|  | TMB | 196 | 196 | 1.15 (0.97, 1.35) | 196 | 196 | 1.16 (0.98, 1.36) | 208 | 208 | 1.03 (0.9, 1.18) |

^a^ Patients in case group who died from unknown or other causes than breast cancer were excluded, together with the matched control patients.

^b^ Patients who had neoadjuvant chemotherapy were excluded, together with the matched case or control patients.

^c^ Genes shared by two or more pathways were removed.

^d^ Odds ratios were estimated using logistic regression models and adjusted for cohort membership, age at diagnosis, menopausal status at diagnosis, molecular subtype, and cancer stage.

# Table S6. The associations between neuroendocrine pathway gene expression in tumor and normal breast tissue and prognosis, stratification analysis by ER status

|  | Tumor (WCH + TCGA) | | Tumor (WCH) | | Tumor (TCGA) | | Normal (WCH) | |
| --- | --- | --- | --- | --- | --- | --- | --- | --- |
| Subgroup | ER+ | ER- | ER+ | ER- | ER+ | ER- | ER+ | ER- |
| Number of patients | 263 | 144 | 109 | 76 | 154 | 68 | 88 | 54 |
| P values ^a^ |  |  |  |  |  |  |  |  |
| Adrenergic | 0.045 | 0.31 | 0.049 | 0.401 | 0.116 | 0.746 | 0.825 | 0.472 |
| Glucocorticoid | 0.016 | 0.077 | 0.031 | 0.623 | 0.044 | 0.268 | 0.683 | 0.429 |
| Dopaminergic | 0.029 | 0.273 | 0.028 | 0.396 | 0.109 | 0.749 | 0.781 | 0.672 |
| Serotonergic | 0.015 | 0.114 | 0.014 | 0.411 | 0.067 | 0.604 | 0.799 | 0.418 |
| Cholinergic | 0.052 | 0.278 | 0.05 | 0.541 | 0.056 | 0.625 | 0.559 | 0.651 |

^a^ P value was calculated using Global test, adjusted for cohort membership, age at diagnosis, menopausal status at diagnosis, molecular subtype, and cancer stage.

# Table S7. The associations between neuroendocrine pathway gene expression in tumor tissue and prognosis, in subsets of the cohorts or exclusive gene list

|  | Exclude non-breast-cancer death ^a^ | Exclude neoadjuvant chemotherapy ^b^ | Remove overlapping genes ^c^ |
| --- | --- | --- | --- |
| Number of patients | 383 | 383 | 407 |
| P values ^d^ |  |  |  |
| Adrenergic | 0.115 | 0.088 | 0.059 |
| Glucocorticoid | 0.04 | 0.023 | 0.063 |
| Dopaminergic | 0.174 | 0.184 | 0.499 |
| Serotonergic | 0.012 | 0.014 | 0.035 |
| Cholinergic | 0.122 | 0.114 | 0.069 |

^a^ Patients in case group who died from unknown or other causes than breast cancer were excluded, together with the matched control patients.

^b^ Patients who had neoadjuvant chemotherapy were excluded, together with the matched case or control patients.

^c^ Genes shared by two or more pathways were removed.

P value was calculated using Global test, adjusted for cohort membership, age at diagnosis, menopausal status at diagnosis, molecular subtype, and cancer stage.

# Table S8. The full list of genes of the glucocorticoid pathway expressed in tumor tissue associated with mutation and prognosis

| Symbol | Glucocorticoid Pathway TMB | | Breast cancer iDFS | |
| --- | --- | --- | --- | --- |
|  | Beta and 95% CIs | P value | Odds ratio and 95% CIs | P value |
| *FOS* | -0.47(-0.76,-0.17) | 0.002 | 0.99(0.85,1.14) | 0.845 |
| *HSP90AA1* | 0.22(0.07,0.36) | 0.003 | 1.5(1.1,2.04) | 0.010 |
| *ADCY4* | -0.29(-0.48,-0.1) | 0.003 | 0.79(0.63,0.99) | 0.045 |
| *KCNJ6* | 0.25(0.09,0.41) | 0.003 | 1.18(0.9,1.54) | 0.228 |
| *GMEB2* | 0.13(0.04,0.22) | 0.004 | 1.19(0.74,1.92) | 0.467 |
| *SLC12A5* | 0.21(0.06,0.35) | 0.005 | 0.72(0.53,0.98) | 0.034 |
| *GNG11* | -0.28(-0.48,-0.09) | 0.005 | 0.91(0.73,1.13) | 0.390 |
| *MMP13* | 0.53(0.11,0.95) | 0.013 | 1.07(0.97,1.19) | 0.183 |
| *HSP90AB1* | 0.16(0.03,0.28) | 0.015 | 1.25(0.89,1.76) | 0.201 |
| *GNB5* | -0.14(-0.26,-0.02) | 0.025 | 1.14(0.79,1.62) | 0.484 |
| *GLUL* | -0.18(-0.34,-0.02) | 0.027 | 0.84(0.63,1.1) | 0.203 |
| *GNG7* | -0.23(-0.43,-0.02) | 0.029 | 0.75(0.6,0.93) | 0.010 |
| *ELK1* | 0.1(0.01,0.19) | 0.031 | 0.97(0.6,1.56) | 0.895 |
| *SLC38A3* | 0.29(0.02,0.55) | 0.033 | 0.95(0.81,1.12) | 0.550 |
| *SRC* | 0.15(0.01,0.28) | 0.033 | 0.86(0.62,1.19) | 0.370 |
| *XIAP* | 0.1(0.01,0.2) | 0.034 | 1.5(0.95,2.37) | 0.083 |
| *MAPK13* | 0.15(0.01,0.3) | 0.039 | 1.12(0.83,1.51) | 0.448 |
| *UBE2I* | 0.1(0,0.2) | 0.045 | 1.11(0.71,1.73) | 0.655 |
| *SMAD4* | -0.1(-0.19,0) | 0.045 | 1.13(0.72,1.78) | 0.598 |
| *GPHN* | -0.15(-0.3,0) | 0.047 | 0.8(0.6,1.07) | 0.130 |
| *JUNB* | -0.19(-0.38,0) | 0.048 | 1.06(0.84,1.33) | 0.651 |
| *CACNA1B* | 0.19(0,0.38) | 0.049 | 1.1(0.87,1.39) | 0.421 |
| *GTF2E1* | 0.09(0,0.19) | 0.050 | 1.1(0.68,1.79) | 0.689 |
| *GNG5* | 0.12(0,0.24) | 0.056 | 0.95(0.67,1.35) | 0.773 |
| *SLC6A11* | 0.24(-0.01,0.49) | 0.063 | 1.16(0.97,1.39) | 0.094 |
| *SLC22A1* | 0.06(-0.01,0.13) | 0.072 | 1.05(0.55,2) | 0.882 |
| *STAT5B* | -0.11(-0.24,0.01) | 0.077 | 0.65(0.45,0.94) | 0.021 |
| *JUND* | -0.15(-0.32,0.02) | 0.089 | 0.76(0.58,0.98) | 0.036 |
| *PTGES3* | 0.09(-0.01,0.19) | 0.091 | 1.31(0.84,2.04) | 0.226 |
| *GNB2* | 0.1(-0.02,0.23) | 0.100 | 1.04(0.73,1.47) | 0.834 |
| *GLS* | 0.12(-0.02,0.27) | 0.103 | 1.25(0.92,1.68) | 0.152 |
| *GNAI3* | 0.07(-0.02,0.16) | 0.111 | 1.61(0.98,2.67) | 0.063 |
| *GLCCI1* | -0.13(-0.28,0.03) | 0.112 | 1.07(0.81,1.4) | 0.655 |
| *SLC38A2* | 0.1(-0.02,0.22) | 0.112 | 1.16(0.81,1.67) | 0.405 |
| *GLS2* | -0.06(-0.14,0.02) | 0.118 | 1.3(0.75,2.26) | 0.355 |
| *TRAK2* | 0.1(-0.03,0.23) | 0.122 | 1.1(0.78,1.55) | 0.572 |
| *STAT5A* | -0.13(-0.3,0.03) | 0.122 | 0.67(0.51,0.88) | 0.004 |
| *TBP* | 0.06(-0.02,0.14) | 0.128 | 1.81(1.03,3.15) | 0.038 |
| *GNB4* | 0.13(-0.04,0.3) | 0.138 | 1.06(0.82,1.37) | 0.667 |
| *GNG2* | -0.13(-0.3,0.04) | 0.142 | 0.84(0.65,1.08) | 0.172 |
| *GNG12* | -0.12(-0.28,0.04) | 0.145 | 1.45(1.09,1.93) | 0.012 |
| *GNG8* | -0.03(-0.07,0.01) | 0.150 | 0.34(0.1,1.12) | 0.076 |
| *SGK1* | -0.12(-0.29,0.05) | 0.156 | 0.93(0.71,1.2) | 0.567 |
| *CACNA1C* | -0.13(-0.31,0.05) | 0.162 | 0.99(0.78,1.27) | 0.943 |
| *CREB5* | -0.14(-0.33,0.06) | 0.166 | 0.97(0.77,1.21) | 0.766 |
| *MAPK1* | 0.07(-0.03,0.16) | 0.171 | 1.59(0.99,2.55) | 0.054 |
| *GABRB2* | 0.11(-0.05,0.27) | 0.187 | 1.55(1.16,2.07) | 0.003 |
| *SLC6A12* | 0.1(-0.05,0.24) | 0.190 | 1.49(1.08,2.04) | 0.014 |
| *CREBBP* | 0.07(-0.04,0.18) | 0.192 | 1.02(0.68,1.54) | 0.912 |
| *GNAI2* | -0.07(-0.17,0.04) | 0.198 | 0.78(0.51,1.19) | 0.247 |
| *GTF2E2* | 0.07(-0.04,0.17) | 0.199 | 0.98(0.65,1.48) | 0.916 |
| *MAPK12* | -0.13(-0.32,0.07) | 0.203 | 1.09(0.87,1.37) | 0.429 |
| *CACNA1F* | -0.1(-0.26,0.06) | 0.209 | 0.84(0.64,1.11) | 0.225 |
| *MAPK11* | -0.11(-0.29,0.07) | 0.221 | 0.88(0.69,1.14) | 0.337 |
| *GNG13* | 0.09(-0.06,0.24) | 0.232 | 0.93(0.69,1.24) | 0.617 |
| *NR3C1* | -0.1(-0.26,0.07) | 0.238 | 0.85(0.65,1.11) | 0.221 |
| *GNB3* | -0.06(-0.17,0.04) | 0.243 | 1.2(0.81,1.79) | 0.369 |
| *ADCY3* | -0.09(-0.24,0.06) | 0.253 | 1.07(0.79,1.44) | 0.669 |
| *ADCY1* | -0.19(-0.52,0.14) | 0.256 | 1.05(0.92,1.19) | 0.499 |
| *GTF2A1* | 0.06(-0.04,0.15) | 0.256 | 1.25(0.79,1.97) | 0.333 |
| *GABRD* | 0.1(-0.07,0.26) | 0.263 | 1.19(0.92,1.55) | 0.189 |
| *MAPK8* | 0.06(-0.04,0.16) | 0.274 | 1.27(0.82,1.96) | 0.285 |
| *POU2F1* | 0.06(-0.05,0.17) | 0.285 | 0.84(0.56,1.25) | 0.387 |
| *JUN* | -0.1(-0.3,0.09) | 0.294 | 1.01(0.8,1.27) | 0.949 |
| *NCOA1* | 0.06(-0.05,0.18) | 0.301 | 0.9(0.62,1.32) | 0.597 |
| *PRKACA* | 0.05(-0.04,0.14) | 0.311 | 0.9(0.56,1.45) | 0.666 |
| *GABARAPL1* | -0.07(-0.21,0.07) | 0.323 | 1.24(0.91,1.68) | 0.174 |
| *GABARAP* | -0.06(-0.18,0.06) | 0.324 | 1.05(0.73,1.52) | 0.788 |
| *FOXO3* | 0.06(-0.07,0.2) | 0.343 | 1.07(0.77,1.48) | 0.696 |
| *CREB1* | 0.04(-0.05,0.13) | 0.366 | 1.63(0.99,2.69) | 0.057 |
| *PRKACB* | -0.11(-0.36,0.14) | 0.376 | 1.14(0.95,1.36) | 0.168 |
| *PRKCB* | -0.12(-0.38,0.15) | 0.383 | 0.82(0.7,0.98) | 0.024 |
| *POU2F2* | -0.08(-0.26,0.1) | 0.384 | 0.72(0.56,0.92) | 0.008 |
| *CREB3* | -0.05(-0.16,0.06) | 0.394 | 1.05(0.71,1.54) | 0.814 |
| *GABBR1* | -0.07(-0.24,0.1) | 0.401 | 1.02(0.78,1.32) | 0.896 |
| *GNB1* | 0.04(-0.05,0.13) | 0.411 | 1.73(1.08,2.79) | 0.024 |
| *HSPA1A* | -0.08(-0.28,0.12) | 0.414 | 1.02(0.82,1.27) | 0.861 |
| *ADCY7* | 0.09(-0.12,0.3) | 0.418 | 1.2(0.97,1.48) | 0.087 |
| *ABAT* | -0.11(-0.37,0.16) | 0.425 | 0.96(0.81,1.13) | 0.601 |
| *CACNA1D* | -0.11(-0.38,0.16) | 0.429 | 0.94(0.8,1.1) | 0.420 |
| *CREB3L4* | 0.08(-0.12,0.28) | 0.438 | 0.96(0.77,1.19) | 0.679 |
| *HSPA4* | 0.04(-0.07,0.15) | 0.455 | 1.3(0.86,1.97) | 0.213 |
| *PLCL1* | -0.08(-0.29,0.13) | 0.457 | 1.07(0.87,1.31) | 0.531 |
| *ADCY5* | -0.13(-0.46,0.21) | 0.464 | 0.85(0.74,0.97) | 0.014 |
| *GNGT2* | -0.04(-0.14,0.07) | 0.477 | 0.62(0.41,0.96) | 0.032 |
| *KAT2B* | -0.05(-0.19,0.09) | 0.485 | 1(0.74,1.35) | 0.997 |
| *SLC38A5* | 0.08(-0.14,0.29) | 0.488 | 1.03(0.84,1.26) | 0.776 |
| *SUMO1* | 0.03(-0.06,0.12) | 0.488 | 0.98(0.61,1.6) | 0.948 |
| *CACNA1A* | 0.05(-0.1,0.2) | 0.505 | 0.92(0.69,1.23) | 0.571 |
| *GTF2F1* | -0.03(-0.13,0.06) | 0.506 | 1.07(0.68,1.69) | 0.758 |
| *SLC38A1* | 0.08(-0.15,0.31) | 0.514 | 0.97(0.8,1.17) | 0.755 |
| *GABARAPL2* | -0.03(-0.14,0.07) | 0.524 | 1.52(0.98,2.34) | 0.059 |
| *GTF2H4* | -0.02(-0.09,0.05) | 0.535 | 0.82(0.44,1.56) | 0.552 |
| *HAP1* | -0.05(-0.21,0.11) | 0.536 | 0.79(0.6,1.05) | 0.105 |
| *MAPK14* | 0.03(-0.06,0.12) | 0.554 | 1.58(0.96,2.59) | 0.071 |
| *GTF2F2* | -0.03(-0.14,0.08) | 0.575 | 1.6(1.05,2.44) | 0.027 |
| *GAD1* | -0.06(-0.28,0.16) | 0.580 | 0.98(0.81,1.2) | 0.874 |
| *PRKCA* | 0.05(-0.13,0.24) | 0.583 | 0.95(0.75,1.21) | 0.679 |
| *GABRP* | 0.14(-0.38,0.66) | 0.597 | 0.96(0.89,1.05) | 0.378 |
| *GTF2H5* | 0.03(-0.09,0.15) | 0.597 | 1.09(0.76,1.56) | 0.641 |
| *SMAD3* | -0.03(-0.17,0.1) | 0.601 | 0.9(0.64,1.25) | 0.523 |
| *GABRE* | -0.09(-0.43,0.25) | 0.608 | 0.9(0.79,1.03) | 0.129 |
| *FKBP4* | 0.05(-0.13,0.22) | 0.613 | 1.02(0.8,1.31) | 0.851 |
| *GNAO1* | -0.05(-0.3,0.19) | 0.666 | 0.91(0.77,1.09) | 0.314 |
| *ADCY9* | -0.03(-0.19,0.13) | 0.689 | 0.95(0.72,1.25) | 0.692 |
| *GTF2H3* | 0.02(-0.08,0.11) | 0.694 | 1.9(1.18,3.05) | 0.008 |
| *GNG4* | 0.06(-0.26,0.38) | 0.706 | 0.94(0.82,1.08) | 0.405 |
| *CEBPB* | -0.03(-0.21,0.15) | 0.714 | 0.94(0.74,1.2) | 0.644 |
| *CDKN1A* | -0.03(-0.22,0.15) | 0.733 | 1.22(0.96,1.56) | 0.098 |
| *NCOA2* | 0.02(-0.13,0.17) | 0.781 | 1.15(0.86,1.53) | 0.359 |
| *NFKB1* | 0.01(-0.09,0.12) | 0.800 | 1.48(0.97,2.25) | 0.069 |
| *CEBPA* | -0.02(-0.21,0.17) | 0.804 | 0.89(0.7,1.12) | 0.321 |
| *GABRB3* | 0.03(-0.27,0.34) | 0.832 | 1.08(0.94,1.25) | 0.291 |
| *HSPA1B* | 0.02(-0.16,0.19) | 0.837 | 1.15(0.9,1.48) | 0.272 |
| *GTF2A2* | 0.01(-0.1,0.12) | 0.846 | 0.94(0.63,1.4) | 0.754 |
| *SERPINE1* | -0.03(-0.29,0.24) | 0.850 | 1.22(1.03,1.44) | 0.024 |
| *NSF* | -0.01(-0.13,0.11) | 0.879 | 1.05(0.73,1.53) | 0.781 |
| *TGFB1* | 0.01(-0.13,0.14) | 0.911 | 0.95(0.69,1.31) | 0.742 |
| *GTF2B* | 0(-0.07,0.08) | 0.914 | 1.57(0.89,2.77) | 0.123 |
| *ADCY2* | -0.02(-0.32,0.28) | 0.915 | 1(0.86,1.15) | 0.947 |
| *GABRQ* | 0.01(-0.26,0.29) | 0.928 | 0.93(0.8,1.09) | 0.391 |
| *GTF2H2* | -0.01(-0.16,0.15) | 0.933 | 1.08(0.81,1.43) | 0.594 |
| *GNAI1* | -0.01(-0.22,0.2) | 0.937 | 0.87(0.71,1.07) | 0.198 |
| *GABRR2* | 0(-0.08,0.08) | 0.943 | 1.08(0.63,1.84) | 0.779 |
| *ADCY6* | 0(-0.15,0.14) | 0.959 | 1.01(0.74,1.38) | 0.953 |
| *GMEB1* | 0(-0.07,0.07) | 0.965 | 1.74(0.91,3.34) | 0.095 |
| *SLC6A1* | 0(-0.13,0.14) | 0.977 | 1.29(0.93,1.78) | 0.130 |
| *GTF2H1* | 0(-0.09,0.08) | 0.980 | 1.58(0.92,2.69) | 0.095 |
| *NFKBIA* | 0(-0.12,0.12) | 0.987 | 0.56(0.38,0.82) | 0.003 |

# Supplementary Methods

Abbreviation: ER, estrogen receptor; PR, progesterone receptor; HER2, human epidermal growth factor receptor 2; IHC, immunohistochemistry; FISH, fluorescence in situ hybridization

# Matching of cases and controls

First, we classified molecular subtype using a surrogate definition of intrinsic subtypes based on the IHC and FISH results of tumor pathology, formulated in St.Gallen consensus in 2013 [1] as follows:

1) Luminal A: ER and PR positive, HER2 negative, and Ki-67 ‘low’.

2) Luminal B

① Luminal B1 (HER2 negative): ER-positive, HER2 negative, and at least one of: Ki-67 ‘high’ or PR ‘negative or low’.

② Luminal B2 (HER2 positive): ER-positive, HER2 over-expressed or amplified, any Ki-67, and any PR.

3) HER2-enriched: HER2 over-expressed or amplified, ER and PR absent.

4) Triple-negative: ER and PR absent, and HER2 negative.

In the WCH cohort, the cut-off is 14% for Ki-67 ‘high’ and 20% for PR positive; HER2 status was determined based on combined information of IHC and FISH.

Next, cases and controls were matched on molecular subtypes defined above in each cohort separately. For the cases with unknown status of HER2, we matched on ER and PR status. Patients with unknown HER2 status were removed from the controls due to incompleteness. One control per case was randomly selected based on these matching criteria.

# Whole exome sequencing and data processing

In TCGA cohort, DNA from tumor tissue was extracted using AllPrep kit (Qiagen). WES was performed using Agilent SureSelect All Exome v2.0 kit or Nimblegen SeqCap EZ Human Exome v2.0 on Illumina Hi-Seq 2000 platform at the Washington University Genome Institute. Somatic mutation data were downloaded from GDC <https://portal.gdc.cancer.gov/files/995c0111-d90b-4140-bee7-3845436c3b42> and a detailed pipeline of variant calling can be found at: <https://docs.gdc.cancer.gov/Data/Bioinformatics_Pipelines/DNA_Seq_Variant_Calling_Pipeline/>.

In the WCH cohort, DNA was extracted from the tumor tissue and germline-derived samples (normal breast tissue, whole blood, or white blood cells) using a Sodium Dodecyl Sulfate (SDS)-based method. We used Agilent Sure Select Human All Exon V6 to prepare sequencing libraries. Whole Exome Sequencing (WES) was performed on Illumina Novaseq S6000 platform, Paired-end 150 in Tianjin Sequencing Center & Clinical Lab of Novogene Co., Ltd.. After quality control, reads were mapped to the reference genome (UCSC hg38) using Burrows-Wheeler Aligner (BWA) software ^29^. Somatic mutations were then called following GATK Best Practices of somatic short variant discovery (SNVs + Indels) (<https://gatk.broadinstitute.org/hc/en-us>). A detailed description of the sample preparation and bioinformatics pipeline for WCH data were available below.

1 Experimental Procedure

1.1 DNA Quantification & Qualification

The quality of isolated genomic DNA was verified by using these two methods in combination:

(1) DNA degradation and contamination were monitored on 1% agarose gels.

(2) DNA concentration was measured by Qubit® DNA Assay Kit in Qubit® 2.0 Flurometer (Invitrogen, USA).

1.2 Library preparation

A total amount of 0.6 μg genomic DNA per sample was used as input material for the DNA sample preparation. Sequencing libraries were generated using *Agilent SureSelect Human All Exon V6* kit (Agilent Technologies, CA, USA) following the manufacturer’s recommendations and index codes were added to each sample.

Briefly, fragmentation was carried out by a hydrodynamic shearing system (Covaris, Massachusetts, USA) to generate 180-280 bp fragments. The remaining overhangs were converted into blunt ends via exonuclease/polymerase activities. After adenylation of 3’ ends of DNA fragments, adapter oligonucleotides were ligated. DNA fragments with ligated adapter molecules on both ends were selectively enriched in a PCR reaction. After PCR reaction, libraries were hybridized with liquid phase with biotin-labelled probes, then magnetic beads with streptomycin were used to capture the exons of genes. Captured libraries were enriched in a PCR reaction to add index tags. Products were purified using the AMPure XP system (Beckman Coulter, Beverly, USA) and quantified using the Agilent high sensitivity DNA assay on the Agilent Bioanalyzer 2100 system.

1.3 Clustering & Sequencing

The clustering of the index-coded samples was performed on a cBot Cluster Generation System using Hiseq PE Cluster Kit (Illumina) according to the manufacturer’s instructions. After cluster generation, the DNA libraries were sequenced on Illumina Novaseq S6000 platform and 150 bp paired-end reads were generated.

2 Bioinformatics Analysis Pipeline

2.1 Quality Control

The original fluorescence image files are transformed to short reads (Raw data) by base calling and these short reads are recorded in FASTQ format, which contains sequence information and corresponding sequencing quality information.

The steps of data processing using trim_galore were as follows:

(1) Trim low-quality ends (<28 Phred score) from reads in addition to adapter removal;

(2) Instructs Cutadapt to use ASCII+33 quality scores as Phred scores (Sanger/Illumina 1.9+ encoding) for quality trimming;

(3) Discard reads that became shorter than length 30 because of either quality or adapter trimming;

(4) Set the tolerable number of bases (equal to 3) that overlap with adapter sequence required to trim a sequence.

All the downstream bioinformatics analyses were based on high-quality clean data, which were retained after these steps. At the same time, QC statistics including total reads number, raw data, raw depth, sequencing error rate and percentage of reads with Q28 (the percent of bases with Phred-scaled quality scores greater than 28) were calculated and summarized.

2.2 Reads Mapping to Reference Sequence

Valid sequencing data were mapped to the reference human genome (UCSC hg38) using Burrows-Wheeler Aligner (BWA) software [2] to get the original mapping results stored in BAM format. If one or one paired read(s) were mapped to multiple positions, the strategy adopted by BWA was to choose the most likely placement. If two or more most likely placements were presented, BWA picked one randomly. Then, using samtools [3] and following GATK practice (https://github.com/broadinstitute/gatk) we transformed SAM files to BAM files, sorted BAM files, marked duplicates, and recalibrated base quality score, generating final BAM file for computation of the sequence coverage and depth.

2.3 Somatic mutation Calling

GATK mutect2 was used to call somatic mutation and identify SNP, InDels. Then we used GATK filtermutectcalls to filter the raw output of Mutect2 in VCF (Variant Call Format).

2.4 Functional Annotation and VCF to MAF

Variant Effect Predictor (VEP) was performed to annotate the VCF file obtained in the previous step. 1000 Genome (https://www.internationalgenome.org/) was applied to characterize the detected variants. Annotation content contained the variant position, variant type, conservative prediction, etc. These annotation results help to locate disease causal mutant. VCF files were then transformed into Mutation Annotation Format (MAF) using vcf2maf [4].

# RNA-sequencing and data processing

In TCGA, RNA from frozen tumor tissue was extracted using AllPrep kit (Qiagen). RNA was analyzed via the RNA6000 nano assay (Agilent) for determination of an RNA Integrity Number (RIN), and only the cases with RIN >7.0 were included in the analysis. Raw read counts produced by HT-Seq were downloaded from the GDC portal. The pipeline was described in detail at: <https://docs.gdc.cancer.gov/Data/Bioinformatics_Pipelines/Expression_mRNA_Pipeline/>.

In the WCH cohort, RNA from frozen tumor and normal breast tissue was extracted using TRIzol Reagent. RNA integrity was assessed using the RNA Nano 6000 Assay Kit of the Agilent Bioanalyzer 2100 system and RIN > 6.0 was required to pass the quality control. RNA sequencing was performed on the Illumina Novaseq S6000 platform, Paired-end 150. After quality control, reads were mapped to reference genome using Hisat2 v2.0.5 ^30^. featureCounts ^31^ was used to count the reads mapped to each gene. A detailed description of the sample preparation and bioinformatic pipeline for WCH data were available below.

1. Sample collection and preparation

1.1 RNA quantification and qualification

RNA degradation and contamination were monitored on 1% agarose gels. RNA purity was checked using the NanoPhotometer® spectrophotometer (IMPLEN, CA, USA). RNA concentration was measured using Qubit® RNA Assay Kit in Qubit® 2.0 Fluorometer (Life Technologies, CA, USA). RNA integrity was assessed using the RNA Nano 6000 Assay Kit of the Bioanalyzer 2100 system (Agilent Technologies, CA, USA).

1.2 Library preparation for Transcriptome sequencing

A total amount of 2 μg RNA per sample was used as input material for the RNA sample preparations. Sequencing libraries were generated using NEBNext® UltraTM RNA Library Prep Kit for Illumina® (NEB, USA) following the manufacturer’s recommendations and index codes were added to attribute sequences to each sample. Briefly, mRNA was purified from total RNA using poly-T oligo-attached magnetic beads. Fragmentation was carried out using divalent cations under elevated temperature in NEBNext First Strand Synthesis Reaction Buffer(5X). The first strand of cDNA was synthesized using random hexamer primer and M-MuLV Reverse Transcriptase (RNase H-). The second strand of cDNA synthesis was subsequently performed using DNA Polymerase I and RNase H. Remaining overhangs were converted into blunt ends via exonuclease/polymerase activities. After adenylation of 3’ ends of DNA fragments, NEBNext Adaptor with hairpin loop structure was ligated to prepare for hybridization. To select cDNA fragments of preferentially 150~200 bp in length, the library fragments were purified with AMPure XP system (Beckman Coulter, Beverly, USA). Then adaptor-ligated cDNA at 37°C for 15 min followed by 5 min at 95 °C before PCR. Then PCR was performed with Phusion High-Fidelity DNA polymerase, Universal PCR primers and Index (X) Primer. At last, PCR products were purified (AMPure XP system) and library quality was assessed on the Agilent Bioanalyzer 2100 system.

1.3 Clustering and sequencing

The clustering of the index-coded samples was performed on a cBot Cluster Generation System using TruSeq PE Cluster Kit v3-cBot-HS (Illumina) according to the manufacturer’s instructions. After cluster generation, the library preparations were sequenced on an Illumina Novaseq S6000 platform and 150 bp paired-end reads were generated.

2. Data Analysis

2.1 Quality control

Raw data (raw reads) of fastq format were firstly processed using trim_galore. In this step, clean data (clean reads) were obtained by removing reads containing adapter, reads containing ploy-N and low quality reads from raw data. At the same time, Q20, Q30 and GC content in the clean data were calculated. All the downstream analyses were based on the clean data with high quality.

2.2 Reads mapping to the reference genome

Reference genome and gene model annotation files were downloaded from the genome website directly. Index of the reference genome was built and paired-end clean reads were aligned to the reference genome using Hisat2 v2.0.5 [5], which can generate a database of splice sets based on the gene model annotation file.

2.3 Quantification of gene expression level

featureCounts [6] was used to count the reads mapped to each gene. featureCounts implements highly efficient chromosome hashing and feature blocking techniques. It is considerably faster than existing methods (by an order of magnitude for gene-level summarization) and requires far less computer memory. It works with paired-end reads and provides a wide range of options appropriate for different sequencing applications.

**Reference**

1. Goldhirsch A, Winer EP, Coates AS, Gelber RD, Piccart-Gebhart M, Thürlimann B, et al. Personalizing the treatment of women with early breast cancer: highlights of the St Gallen International Expert Consensus on the Primary Therapy of Early Breast Cancer 2013. Ann Oncol. 2013;24(9):2206.

2. Li H, Durbin R. Fast and accurate short read alignment with Burrows–Wheeler transform. Bioinformatics. 2009;25(14):1754–60.

3. Li H, Handsaker B, Wysoker A, Fennell T, Ruan J, Homer N, et al. The Sequence Alignment/Map format and SAMtools. Bioinformatics. 2009;25(16):2078–9.

4. Kandoth C. mskcc/vcf2maf: vcf2maf v1.6.19. Vol. 2020.

5. Kim D, Paggi JM, Park C, Bennett C, Salzberg SL. Graph-based genome alignment and genotyping with HISAT2 and HISAT-genotype. Nat Biotechnol 2019 378. 2019;37(8):907–15.

6. Liao Y, Smyth GK, Shi W. featureCounts: an efficient general purpose program for assigning sequence reads to genomic features. Bioinformatics. 2014;30(7):923–30.
